# Modular Framework for 3D Molecular Generation in Computational Chemistry Applications

**DOI:** 10.1021/jacs.5c19960

**Published:** 2026-06-22

**Authors:** Thanapat Worakul, Mohammed Azzouzi, Matthew D. Wodrich, Clémence Corminboeuf

**Affiliations:** † Laboratory for Computational Molecular Design, Institute of Chemical Sciences and Engineering, 27218 Ecole Polytechnique Fédérale de Lausanne (EPFL), 1015 Lausanne, Switzerland; ‡ Laboratory for Computational Molecular Design, Institute of Chemical Sciences and Engineering, École Polytechnique Fédérale de Lausanne (EPFL), 1015 Lausanne, Switzerland; § National Center for Competence in Research-Catalysis (NCCR-Catalysis), École Polytechnique Fédérale de Lausanne (EPFL), 1015 Lausanne, Switzerland

## Abstract

Three-dimensional molecular generative models construct molecules by assigning atoms explicit Cartesian coordinates. This formulation allows the molecular generation process to be guided by geometric (e.g., steric effects, molecular shape) and physicochemical constraints toward desired objectives in a given chemical problem. The practical adoption of these models, however, remains limited by two key challenges: the high computational cost associated with model training as well as the lack of standardized platforms that allow models to be developed, evaluated, and compared. The landscape of 3D generative models for molecular design is fragmented, with most methods developed for specific molecular generation objectives. The implementations of these models are scattered across different repositories, software environments, and evaluation protocols, which impedes reproducibility and controlled comparison of different approaches and, generally, limits broader adoption. Here, we introduce MolCraftDiffusion, a modular and extensible platform for building, deploying, and evaluating 3D molecular diffusion models in computational chemistry. Its layered architecture decouples core training logic from model definitions and task implementations, enabling new generative architectures, guidance strategies, and evaluation metrics to be integrated. As part of the package, we implement curriculum learning, a progressive chemical complexity training approach used to construct a pretrained diffusion model on 3D molecular data sets compiled from multiple sources, circumventing the cost of full training in downstream applications. The framework provides a modular set of guidance mechanisms for directing generation toward chemically relevant objectives: structure control via molecular inpainting (systematic exploration of structural variants around a reference) and outpainting (extending molecules with new chemical groups), and property-conditioned generation via gradient-based and classifier-free approaches. We illustrate these capabilities across computational chemistry tasks, including virtual library construction and inverse molecular design. The extensibility of the platform is further demonstrated by porting three architecturally distinct models from the literature: TABASCO, ADiT, and ShEPhERD, each integrated without modifications to the core codebase. The codebase, pretrained models, and examples are available at: https://github.com/lcmd-epfl/MolCraftDiffusion and https://huggingface.co/pregH/MolecularDiffusion.

## Graphical abstract

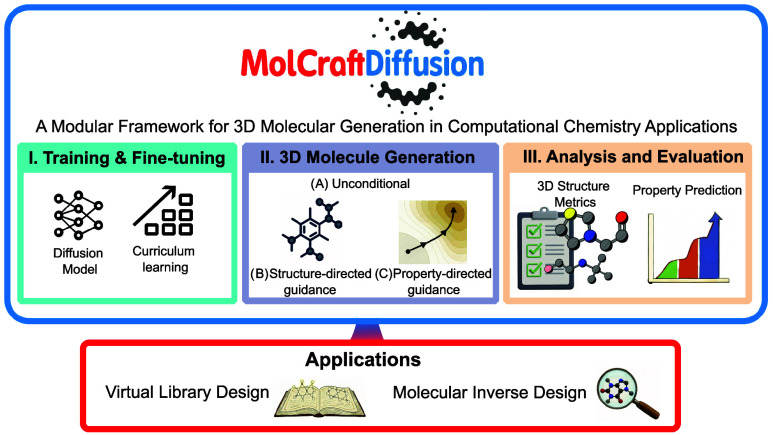

## Introduction

Among AI-based approaches in chemistry, generative deep learning^1–10^ has the potential to facilitate the exploration of chemical space. These models learn the underlying statistical distribution of molecular structures from large datasets, enabling the de novo generation of compounds that resemble those in the training data. This capability not only accelerates the discovery of molecules but also expands molecular repositories in both scope and diversity, thereby strengthening the foundation for data-driven molecular design and property prediction.

Most existing molecular generative models consider either string-based representations (e.g., SMILES)^11–22^ or graph-based molecular representations.^23–31^ The models capable of directly generating three-dimensional (3D) molecular geometries are relatively less established owing to the inherent complexity of 3D conformational space, spatial constraints, and molecular symmetry. Nevertheless, since most molecular properties depend on 3D structure, direct 3D generation provides a more physically meaningful route for molecular discovery. By conditioning the generative process directly in Cartesian space, 3D molecular generative models enable the design of candidates that satisfy both electronic and geometric requirements, an essential capability for applications such as catalysis^32–35^ and protein–ligand binding.^36–40^

For 3D molecular structure generation, diffusion models,^41–44^ which learn to reverse a gradual noising process to sample complex data distributions, have emerged as one of the most promising generative frameworks. One of the first applications of diffusion models to 3D molecular systems was introduced by Hoogeboom et al.^45^ which used E(3)-equivariant graph neural networks to predict atom types and Cartesian coordinates. While reliable for small organic molecules, its performance deteriorates on larger, structurally complex systems, prompting a succession of more robust architectures that can generate increasingly intricate 3D structures.^46–50^ These advances, while improving the quality of generated molecules, generally raise computational demands for training and generation.

To adapt diffusion models for molecular discovery applications, several guidance diffusion techniques have been developed to steer the generative process toward specific 3D structures or target properties. Structure-directed guidance methods^36,39,40,51–61^ have enabled applications such as scaffold decoration,^58,62^ shape-conditioned generation,^56^ and lead optimization.^53^ In parallel, property-directed guidance approaches,^63–65^ often based on gradient-based or classifier-free guidance, bias generation toward molecules satisfying target electronic or geometric criteria. Despite these advances, existing methods remain largely task-specific and are implemented in isolation, with no common infrastructure for training, evaluation, or systematic comparison across approaches for 3D molecule generation.

To address this gap, we introduce MolCraftDiffusion, a modular and extensible platform for building and deploying 3D molecular diffusion models (Figure 1). The platform provides a unified environment in which different generative architectures and guidance strategies operate under identical training, generation, and evaluation protocols, enabling reproducible workflows and fair comparison across models. New architectures, guidance mechanisms, and evaluation metrics can be incorporated with minimal changes to the core codebase. All workflows, including training, fine-tuning, generation, property regression, and evaluation, are accessible via a command-line interface with YAML configuration files. Within this platform, we develop curriculum learning, a progressive chemical complexity training approach, to construct a pretrained diffusion model on a chemically diverse dataset of 3D molecular structures compiled from multiple sources, circumventing the cost of full training in downstream applications. We further integrate a comprehensive suite of structure-directed and property-directed generation mechanisms and illustrate their utility across molecular generation applications, including virtual library construction and inverse molecular design. The extensibility of the platform is demonstrated by porting three architecturally distinct models from the literature, specifically, TABASCO^66^ (Transformer-based atomistic diffusion model operating under a flow-matching framework), ADiT^48^ (all-atom latent diffusion transformer), and ShEPhERD^67^ (SE(3)-equivariant diffusion for pharmacophore-conditioned generation) on top of the E(3)-equivariant diffusion model (EDM).^45^ Collectively, MolCraftDiffusion provides a common foundation for integrating and comparing current and future advances in 3D molecular generative modeling within data-driven computational chemistry workflows.

**Figure 1:**
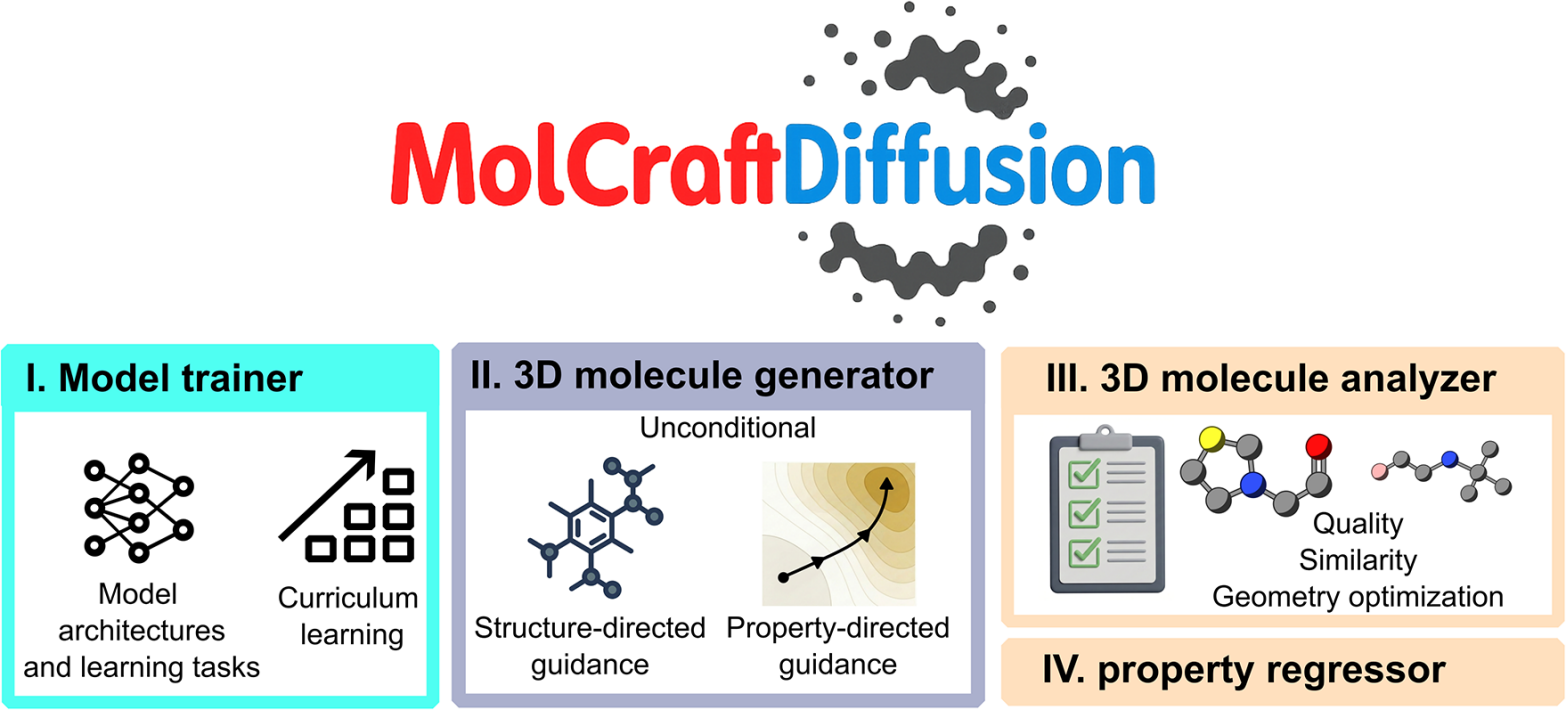
Overview of the MolCraftDiffusion platform, consisting of four modules: (i) *model trainer*, for building 3D molecular diffusion models, property regressors, and guidance models; (ii) *3D molecule generator*, for controlled 3D molecular generation via structure-directed and/or property-directed guidance mechanisms; (iii) *3D molecule analyzer*, for the standardized evaluation of generated 3D molecular structures; and (iv) *property regressor*, for property regression from 3D molecular inputs.

## Methods

MolCraftDiffusion is a modular Python package for building and deploying 3D molecular diffusion models in computational chemistry. The platform is organized into four modules: training, generation, prediction, and analysis (Figure 1). The training module supports training models (i.e., diffusion model, property regressor, and guidance model (time-aware property regressor))­from scratch or fine-tuning pretrained models. The generation module provides a suite of guidance mechanisms distinguished by target type: structure-directed generation, which steers generation toward molecules satisfying specific structural constraints, and property-directed generation, which biases sampling toward molecules with target physicochemical or electronic properties. The prediction and analysis modules enable direct property regression from 3D molecular inputs and standardized evaluation of generated structures, respectively.

In this section, we describe each component of MolCraftDiffusion. We first present the 3D diffusion model and the curriculum learning strategy that improves training efficiency (Figure 2a). We then introduce the guidance mechanisms: structure-directed methods including molecular inpainting and outpainting (Figure 2b), and property-directed methods, including gradient-based and classifier-free guidance (Figure 2c). We next define the structure quality metrics used to assess generative model performance, followed by an overview of the MolCraftDiffusion framework.

**Figure 2:**
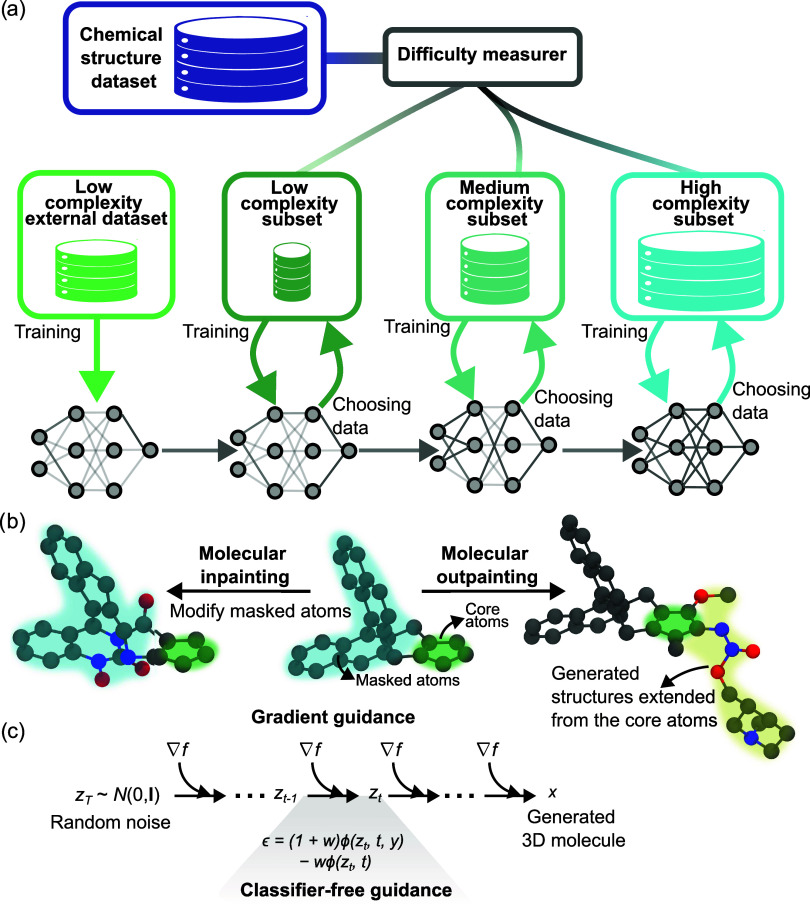
(a) Curriculum learning workflow, where molecular size is used to organize the data set into progressively challenging subsets for training. (b) Structure-directed guidance methods: molecular inpainting, which selectively modifies masked atoms, and molecular outpainting, which generates additional molecular substructures anchored to a predefined core. (c) Property-directed generation methods, including gradient-based guidance, where the gradient ∇*f* is added during each denoising step, and classifier-free guidance, which directs generation toward the target *y* by amplifying the difference between conditional ϕ­(**z**_**t**_, *t*, *y*) and unconditional predictions ϕ­(**z**_**t**_, *t*).

### Diffusion Model for 3D Molecule Generation

The generative model currently used in MolCraftDiffusion is based on the E(3)-equivariant diffusion model (EDM) introduced by Hoogeboom et al.^45^ In this model, a molecule is represented as a set of atom types and their corresponding 3D Cartesian coordinates, ***x*** = (**h**, **x**). The model operates through a diffusion process, which consists of two phases: a forward process that gradually adds Gaussian noise to the molecular structure until it becomes pure noise, and a reverse process that learns to denoise it back to a valid molecule.

The core of the diffusion process is a denoising network, ϕ­(**z**_*t*_, *t*), which predicts the noise added at each time step *t*. We employ an Equivariant Graph Neural Network (EGNN) for this task, which respects the rotational and translational symmetries of 3D molecules. The network is trained by minimizing the mean squared error between the predicted and true noise. Generation starts from random noise and iteratively applies the denoising network to produce a clean molecular structure. Concretely, sampling proceeds by (i) initializing $z_{T}∼N\left(0,I\right)$ ; (ii) for *t* = *T*, ..., 1 with *s* = *t* – 1, computing the Gaussian transition $p\left(z_{s}|z_{t}\right)=N\left(\mu ,\sigma^{2}I\right)$ with $\mu =\frac{1}{\alpha_{t|s}}z_{t}-\frac{\sigma_{t|s}^{2}}{\alpha_{t|s}\sigma_{t}}\phi \left(z_{t},t\right)$ and $\sigma =\sigma_{t|s}\frac{\sigma_{s}}{\sigma_{t}}$ , then sampling **z**_*s*_; (iii) after each update, subtracting the center of gravity of the coordinate block **z**_*s*_^(*x*)^ in the concatenated latent **z**_*s*_ = [**z**_*s*_^(*x*)^, **z**_*s*_^(*h*)^] to preserve translation equivariance; and (iv) decoding **z**_0_ to obtain [**x**, **h**]. This procedure corresponds to Algorithm 1 and uses a variance-preserving noise schedule to define α_*t*_, σ_*t*_, and their stepwise ratios α_*t*|*s*_ and σ_*t*|*s*_. A detailed description of the diffusion equations, model architecture, and generation algorithm is provided in the Supporting Information.
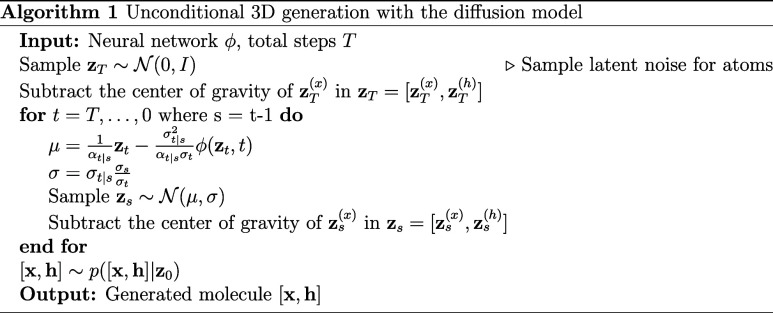


An ablation study of the equivariant diffusion model architecture on the QM9 data set, examining the effects of model capacity, noise scheduling, and atomic feature choices, is provided in Section 9 of Supporting Information.

### Training with Curriculum Learning

Training these diffusion models can be computationally demanding and often difficult to converge owing to the high-variance nature of the denoising objective.^68^ To mitigate these challenges, we complement conventional training with curriculum learning strategies^69–71^ for molecular diffusion models. In these approaches, the model is first trained on simpler molecules and gradually exposed to more structurally complex molecules, facilitating smoother convergence.

We implement three curriculum learning strategies: predefined curriculum learning (PCL),^69^ self-paced learning (SPL), and hybrid curriculum learning (HCL). PCL follows a fixed curriculum, where molecules are ordered according to predefined difficulty criteria such as molecular size, number of heavy atoms, or topological complexity (e.g., presence of rings or branching). This approach ensures a structured and interpretable training process, making it straightforward to implement and analyze.

SPL instead allows the model to dynamically select training samples based on loss magnitude (Figure 3b). Starting from a model pretrained on small molecules, we apply a hard regularizer^72^ that discards molecules in each batch whose loss exceeds a predefined threshold. This threshold is gradually increased over training, ensuring the model first learns from simpler molecules before progressing to more complex structures. This adaptive mechanism provides flexibility and can improve the learning convergence.

**Figure 3:**
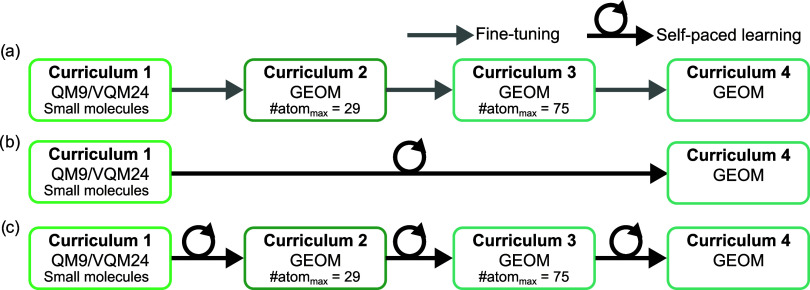
Curriculum learning strategies employed in this work including (a) PCL, where subsets are manually selected based on predefined complexity criteria and trained sequentially. (b) SPL, where the model autonomously selects training samples based on difficulty. (c) HCL, integrating SPL with PCL, enabling adaptive progression while utilizing predefined data set partitions.

Finally, we combine both approaches into the HCL strategy: the data set is first ordered using the predefined molecular size-based criterion, and self-paced learning is employed at each stage (Figure 3c). This hybrid strategy leverages the interpretability of a fixed curriculum while retaining the adaptability of SPL, enabling the model to handle unanticipated complexity not captured by predefined criteria.

### Guidance Generation Approaches

With diffusion models now capable of generating 3D molecular structures, an important next step is to guide the denoising process toward molecules that exhibit specific attributes relevant for downstream applications. In the following, we categorize and describe these guidance methods according to the nature of the target attributes: geometrical/structural constraints or molecular properties.

#### Structure Guidance Diffusion

To direct the denoising generative process to molecules with target geometrical or structural attributes, we consider inpainting and outpainting approaches. While several existing approaches incorporate structural constraints into the molecular generation,^36,39,40,51–61^ they are often embedded directly into the model architecture and require specialized training objectives and datasets. In contrast, our proposed guidance methods operate in a plug-and-play manner, allowing integration with the unconditionally trained diffusion models.

Inpainting enables systematic modification of a reference 3D molecular structure.^73^ This technique was originally developed for image restoration and completion tasks.^74,75^ For 3D molecules, inpainting involves applying noise at a specified level *d* (denoising strength) to the atomic attributes and positions, thereby partially removing structural information (Algorithm 2). The denoising process is then carried out from the noise level *d* onward to generate new 3D molecules. Higher *d* values result in higher variation from the reference 3D molecule. In contrast, lower *d* values allow more structural information from the reference to be retained, thereby encouraging the generation of molecules that resemble the original 3D structure. Our implementation of molecular inpainting also allows for targeted modifications by applying noise only to a designated subset of atoms (i.e., the masked atoms), while the remaining unmasked atoms are kept fixed throughout the denoising process. These capabilities are particularly useful for applications such as fragment replacement^62^ or side-chain decoration,^76^ enabling precise edits to specific regions of a molecule.
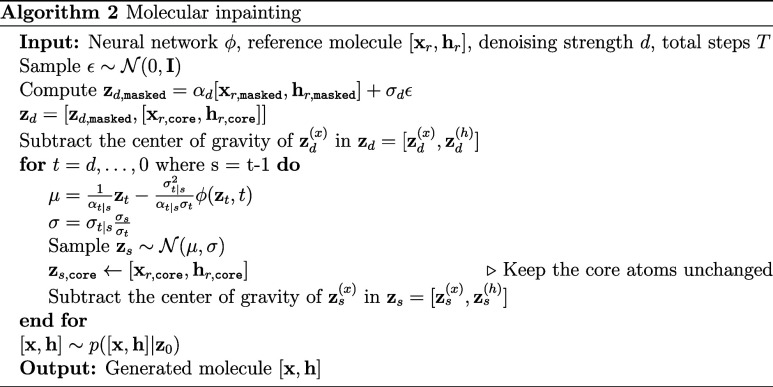


Outpainting, on the other hand, incorporates the target 3D core structure into the generated molecules. Outpainting is a method used for image extrapolation tasks.^73,74^ This method conditions the diffusion model to generate 3D molecular structures around a fixed core structure. With this capability, molecular outpainting not only enables scaffold decoration^58,62^ for in silico library design but also facilitates goal-directed generation when specific geometrical attributes of the molecules are the design targets.

In the outpainting, the initial latent variable at the final time step *T*, **z**_*T*_, is formed by concatenating the core structure with randomly initiated atoms. During the denoising process, the coordinates and atom types of the core part are kept fixed. To prevent steric clashes, we also enforce spatial constraints to ensure that atoms in the generated region do not overlap with the core scaffold.
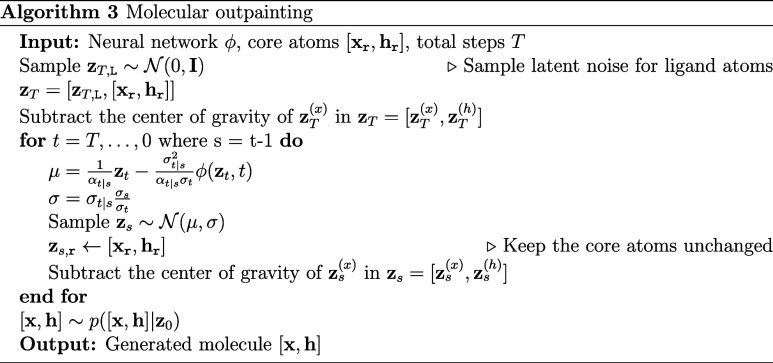


While outpainting can be applied directly in a plug-and-play manner with pretrained diffusion models, we introduce two additional strategies to improve generation quality. First, during the denoising generative process, we allow the denoising of all atom positions in the final steps of the denoising generative process while keeping the core atom types fixed. This late-stage relaxation allows the core to adjust spatially and better integrate with the generated structure. Second, we propose a dedicated conditional training objective tailored for molecular outpainting. In this training scheme, the positions and atom types of the core atoms are frozen during the forward process. Consequently, the model is trained to ignore these core atoms and predict the noise only for the remaining, noncore atoms, explicitly adapting the model to perform the conditional generation task. Instead of training from scratch, MolCraftDiffusion enables fine-tuning of an unconditionally trained model for this outpainting training objective.
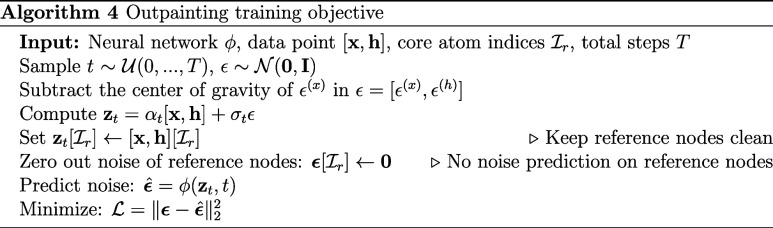


#### Property Guidance Diffusion

In addition to guiding the generative process to molecules with specific structural features, we incorporate two complementary property-conditioning techniques aimed at biasing generation toward molecules with specific target properties *y* (Figure 2c): gradient guidance and classifier-free guidance (CFG).

Gradient guidance, also known as classifier-based guidance,^77^ operates in a plug-and-play fashion. It perturbs the latent variable at each denoising step *t* (**z**_*t*_) using the gradient of a differentiable target function ∇_**z**_*t*__ *f*(**z**_*t*_) that reflects desired properties. This gradient guides the generation process toward the desired property without requiring fine-tuning of the diffusion model. Obtaining such gradients necessitates a target function that is continuous and differentiable with respect to **z**_*t*_ and operates on the same molecular representation, Cartesian coordinates, and node attributes as used in the diffusion model.

To fulfill this requirement, we train a guidance model, *f*_θ_(**z**_*t*_, *t*), capable of predicting the target property *y* directly from the latent representation at each denoising step. The model shares the same EGNN backbone as the diffusion model and is trained via supervised regression on diffusion-perturbed molecular data (training details are provided in the Supporting Information). Specifically, given a clean molecular structure **x**_0_ and its corresponding property *y*, we perturb **x**_0_ with Gaussian noise according to the diffusion noise scheduler at time step *t* to obtain a noisy input **z_t_**. The guidance model is then optimized to minimize the mean squared error between the predicted and true property values.
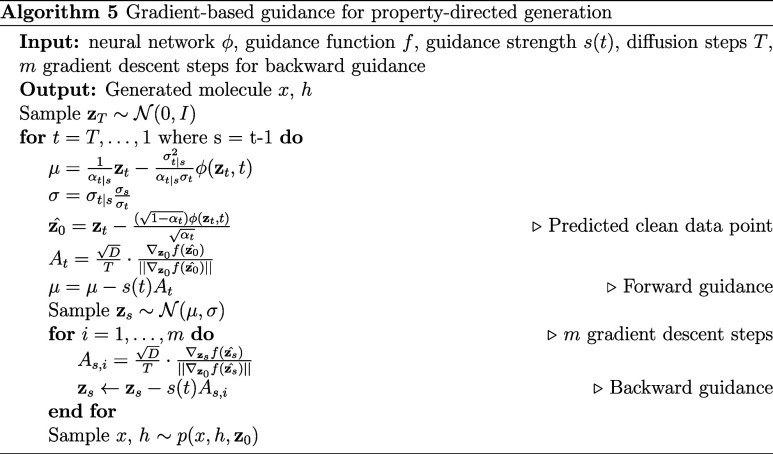


As an alternative to gradient-based schemes, CFG^78^ offers a conceptually different mechanism for incorporating conditioning information. Rather than relying on an external classifier or regressor, CFG trains the diffusion model itself to support both conditional and unconditional generation. Similar to the outpainting training objective, MolCraftDiffusion enables adaptation of an unconditionally trained model for CFG by extending the model with additional input features to accommodate conditioning variables (*y*). To preserve the unconditional mode, these features are randomly dropped with a defined probability (typically 0.1–0.2) by replacing their values with a predefined null token. The training objective otherwise remains unchanged from the unconditional setting.

During inference, the model’s predictions under both conditions are combined to guide the generation with the condition *y*:

1 $$\mathrm{\epsilon ̃}=\left(1+w\right)\phi \left(z_{t},t,y\right)-w\phi \left(z_{t},t\right)$$

Here, ϕ­(**z**_**t**_, *t*, *y*) represents the noise prediction from the conditional model (with property *y* provided) and ϕ­(**z**_**t**_, *t*) corresponds to the unconditional prediction. The CFG scale parameter *w* ⩾ 0 controls the strength of conditioning. By increasing *w*, the generation is more biased toward satisfying the condition *y*,

### Structure Quality Metrics

The quality of generated 3D molecular structures is critical to their practical utility. Generated molecules must satisfy fundamental chemical rules, including correct valencies and geometries consistent with valence shell electron pair repulsion theory. They should also form a single connected structure and approximate stable conformations along the potential energy surface. Without these checks, poorly generated molecules may still satisfy task-specific target criteria but lack chemical relevance, limiting their value. To ensure a comprehensive evaluation, we employ a suite of complementary metrics, each capturing distinct aspects of chemical validity, stability, and structural integrity, to assess the quality of the generated 3D molecules. To ensure a comprehensive evaluation, we employ a suite of complementary metrics that capture distinct aspects of chemical validity, structural stability, and diversity. Detailed definitions of all metrics are provided in the Supporting Information.

We first apply basic checks for valency and connectivity, including **(1) Chemical validity (%)**, **(2) Connected (%)**, and **(3) Chemically valid and connected (%)**. Next, we assess 3D structural plausibility using the PoseBusters sanity checks,^79^ which compare geometrical attributes in the generated 3D molecules to the reference values. Here, we consider **(4) Bond lengths/angles**, **(5) Ring flat**, **(6) Double bond flatness**, **(7) No steric clash**, and **(8) Internal energy**. We then evaluate deviations from optimized geometries through **(9) Average RMSD** (Å) and **(10) Intact chemical topology (%)**, which together reflect geometric accuracy and structural stability after relaxation. Finally, we quantify chemical diversity to ensure broad exploration of chemical space, using **(11) Uniqueness** and **(12) Novelty**, based on pairwise Tanimoto similarity of molecular fingerprints to assess internal diversity and generalization beyond the training data.

### Overview of the Platform

MolCraftDiffusion is a modular Python package that covers the full pipeline of 3D molecular generative modeling, from data ingestion through training, generation, and evaluation. Its layered architecture decouples core training logic from model definitions and task implementations, enabling independent modification of each component. The data layer accepts multimodal molecular data sets in multiple formats, geometric data (XYZ with CSV metadata), Atomic Simulation Environment (ASE) databases, and preprocessed binaries and automatically handles the collation strategies required by graph neural networks and point cloud representations. Native interfaces to RDKit,^80^ Morfeus,^81^ and Cosymblib^82^ allow chemically relevant node features beyond atomic identity to be incorporated directly.

The platform is organized into four modules: (i) *training*, for building diffusion models, property regressors, and guidance models; (ii) *generation*, supporting controlled generative mechanisms; (iii) *prediction*, for direct property regression from 3D inputs; and (iv) *analysis*, providing standardized evaluation tools. This design enables a systematic and reproducible 3D molecule generative workflow application while remaining extensible: new 3D generative architectures, controlled generation strategies, or molecular quality metrics can be integrated with minimal changes to the core codebase. In practice, all modules are accessible via a command-line interface, with tasks specified through YAML configuration files. Tutorials illustrating configuration and usage for each module are publicly available at https://preghosh.github.io/MolCraftDiffusion/. Additional implementation details are provided in Section S10 of the Supporting Information.

## Results and Discussion

### Curriculum Learning for Training Diffusion Models

Ensuring the quality of generated 3D molecular structures is a critical prerequisite for the usability of diffusion models in downstream data-driven applications. To this end, we introduce three curriculum learning strategies for training equivariant diffusion models. Here, we consider the EDM by Hoogeboom et al.^45^ In all curriculum learning cases, the model is first pretrained for 200 epochs on a concatenated dataset of QM9 ^83^ and VQM24,^84^ which contain small-sized organic and inorganic molecules. The pretrained model is then further trained on the GEOM data set^85^ using one of three approaches (PCL, SPL, and HCL) as introduced in the method section.

Conventional training, where we train the diffusion model directly on the GEOM data set, yields a model with poor generation performance, producing only 2.4% valid and connected 3D molecules (Table 1). After geometry optimization with GFN2-xTB, merely 1.1% of these molecules retain the same chemical topology as their optimized counterparts, with an average RMSD of 1.63 Å. Most also fail to pass the PoseBuster tests (Table S2). In contrast, all three curriculum learning strategies substantially improve generation quality (Table 1). The majority of generated molecules are chemically valid, form a single connected graph, and over 90% successfully pass the PoseBuster test. Furthermore, the discrepancies between the generated and optimized counterparts are reduced, with the chemical topology of the generated molecules remaining intact after the geometry optimization, and the RMSD is decreased to ∼ 1 Å.

**Table 1:** Unconditional Generation of 1000 3D Chemical Structures using the EDM Trained With Different Strategies on the GEOM and the Large-Scale Chemical Databases^a^

**model**	chemically valid (%)	connected (%)	valid and connected (%)	RMSD (Å)	intact topology (%)	novelty	uniqueness
**GEOM Database**							
EDM	9.7 ± 0.1	19.3 ± 1.1	2.4 ± 0.2	1.63 ± 0.04	1.1 ± 1.1	**0.72** ± **0.00**	**0.79** ± **0.00**
EDM + PCL	92.3 ± 0.9	76.3 ± 0.8	71.9 ± 0.07	1.08 ± 0.02	83.6 ± 1.5	0.68 ± 0.00	0.78 ± 0.00
EDM + SPL	**95.7** ± **0.9**	79.3 ± 0.7	77.4 ± 1.2	1.00 ± 0.02	86.1 ± 1.0	0.66 ± 0.00	0.76 ± 0.00
EDM + HCL	94.8 ± 1.2	**82.6** ± **1.5**	**79.0** ± **2.1**	**0.97** ± **0.02**	**88.1** ± **2.0**	0.65 ± 0.00	0.76 ± 0.01
**Compiled 3D Molecule Database**							
EDM + HCL	83.4 ± 0.4	82.2 ± 1.9 ±	73.8 ± 1.6	1.03 ± 0.03	83.8 ± 1.4	0.64 ± 0.01	0.77 ± 0.00

a The mean and standard deviation over three trials of these generations are reported.

Among these curriculum learning methods, the HCL, which combines elements of PCL and SPL, delivers the best generative performance (Table 1). The model trained with HCL creates the highest percentage of valid and connected molecules (79.0%). These generated molecules are also the most consistent with their optimized counterparts (RMSD = 0.97 Å, and 88.1% intact chemical topology after geometry optimization). However, while SPL contributes to improved chemical validity and geometric stability, its inclusion slightly compromises uniqueness and novelty compared to models trained with a predefined curriculum alone. This trade-off arises because SPL explicitly discards molecules with high training loss, which can cause the model to learn fewer molecules in the training data.

Building upon the diffusion model trained on the GEOM database, we further fine-tune it with SPL on the compilation dataset of 3D molecules (more information available in the Supporting Information). The resulting pretrained diffusion model generates 73.8% chemically valid and fully connected 3D molecules. Following the geometry optimization, the RMSD between these generated and optimized geometries is 1.03 Å with 83.8% of these molecules retaining their original connectivity. Furthermore, the generated molecules are structurally diverse, both within the batch and relative to the training set, as quantified by a uniqueness score of 0.65 and a novelty score of 0.77.

### 3D Molecular Generative Applications

With a pretrained diffusion model in place, we demonstrate its applicability through structure-directed and property-directed guidance, enabling both virtual library generation and inverse molecular design. In these tasks, we first apply the pretrained model directly with the proposed guidance diffusion methods. If the resulting performance is insufficient, we employ the fine-tuning strategies implemented in MolCraftDiffusion to further adapt the model to these specific downstream applications, thereby enhancing the generative performance. These examples not only illustrate the flexibility of the framework but also serve as case studies to highlight current limitations and to inform avenues for future methodological improvements.

#### Virtual Library Design

We first illustrate the utility of MolCraftDiffusion for constructing virtual libraries of specific molecular families. This is achieved through the use of the structure-guidance methods implemented in the framework, which steer the generative process toward molecules belonging to a desired chemical class. Such libraries not only provide diversified starting points for lead identification, but also define a structured chemical space that can be leveraged for downstream optimization and predictive modeling. As an illustrative case study, a library of asymmetric cyclopentadienyl (Cp) ligands is constructed. Upon complexation with group 9 metals (Co, Rh, Ir), this class of ligands has been shown to facilitate the enantioselective formation of dihydroisoquinolones.^86–89^ Despite their value as important components of transition metal-catalyzed reactions, the complex synthetic effort required to make these ligands has, to the best of our knowledge, precluded the development of extensive screening campaigns. The development of a virtual library, however, could be highly valuable as a database that allows the interplay between sterics and catalytic activity/selectivity to be systematically analyzed. Such knowledge could be used to refine and test ligand design models that go beyond “back-of-the-envelope” with the objective of steering ligand design into an appropriate region of chemical space where species with improved activity/selectivity for a desired reaction can be found.

In building this virtual library of Cp ligands, focus is placed on introducing various amounts of steric diversity at multiple locations within the ligand. As a starting template, we consider 1,1′-bi-2-naphthol cyclopentadienyl (BINOL-Cp) ligand, a widely used asymmetric scaffold in Cp ligands. This ligand’s underlying design principle^90,91^ aims to orient the incoming substrate in a particular manner (Figure 4a) via the manipulation of three key structural components (the back wall, the side wall, and the ceiling) in order to induce high enantioselectivity. These structural components shape the chiral pocket as follows: the back wall hinders access to one face of the catalyst, while the side wall and the ceiling orient the incoming substrate to minimize unfavorable steric interactions (Figure 4a). Here, the BINOL moiety serves as the back and side walls, while selective substitution at the R^1^, R^2^, and R^3^ positions on the Cp ring allows the ceiling to be adjusted. The simplest way of analyzing the steric environment imparted by the backbone as well as these substitutions is by examining the occupancy of the various quadrants and octants around the catalyst’s metal center (Figure 4b). To do this, we examine the buried volume with a radius of 5 Å. Using this quadrant/octant model, the BINOL backbone should primarily dictate the steric environments of Q2 and Q3, with Q3 being occupied and Q2 is unoccupied by the upward and downward pointing nature of the naphthyl moieties. At the same time, structural elements connected to the Cp moiety will vary the occupations of Q1, Q4, O1, and O4, which represent the regions of space where the substrate approaches the metal center during the reaction (Figure 4).^89^ To explore the different steric design spaces intrinsic to these asymmetric ligands, we constructed two libraries, the first of which exclusively makes substitutions on the Cp ring while the BINOL backbone is left untouched, and the second diversifies the BINOL backbone itself. A summary of the workflow for generating these Cp ligands is provided in Figure 5.

**Figure 4:**
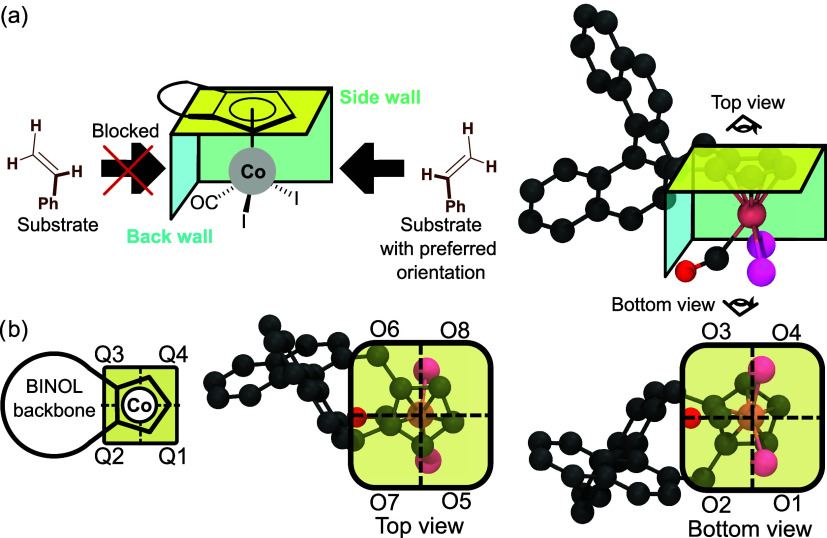
(a) Stereochemical model of the BINOL-Cp catalyst used for steric analysis. The backwall (blue) and sidewall (green) planes define key steric regions around the metal center. (b) The quadrant and octant analysis around the metal center. The steric environment is quantified within a 5 Å buried volume sphere, subdivided into quadrants (Q1–Q4) and octants (O1–O8).

**Figure 5:**
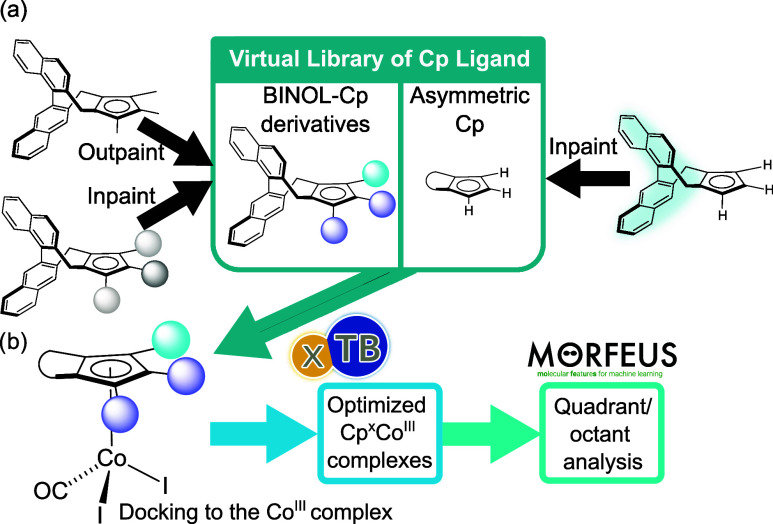
(a) Workflow for generating the virtual library of the asymmetric Cp ligand. (b) Docking of the generated Cp ligands onto a Co­(III) center, followed by geometry optimization using *x*TB. The resulting CpCo­(III) complexes are analyzed with MORFEUS to extract quantitative steric descriptors for downstream modeling.

For the BINOL-Cp derivative set, we start from a bare BINOL-Cp scaffold and apply molecular outpainting, a technique that generates atoms conditioned on a given scaffold. In this case, the method completes the BINOL-Cp framework by generating substituents at the R^1^, R^2^, and R^3^ positions (Figure 5a). Among the initial 2,500 structures generated with outpainting, 745 of these pass the valency check and possess an overall charge of −1, qualifying them as chemically (although not necessarily synthetically) valid Cp ligands (success rate = 29.8%). To assess the steric properties of these ligands, each valid Cp ligand is first docked onto a Co­(III) complex,^92^ and then optimized at the GFN2-xTB level. Following optimization, a set of steric descriptors for the Cp ligands is computed using the Morfeus package^81^ (Figure 5b).

These generated BINOL-Cp derivatives span a broad region of the O1/O4 property map, in contrast to existing experimentally realized Cp ligands, which are predominantly clustered in the bottom-left region, indicative of relatively unhindered steric environments (Figure 6b,c). Yet, this initial pool of generated structures still only sparsely populates the top-right region of the map (Figure 6b), with most ligands remaining in the lower-left cluster. To enrich this library and cover the missing area of the O1/O4 map, we selected two ligands (**1** and **2**) located proximate to the uncovered area, and employed the inpainting (as opposed to the previously employed outpainting) method to vary their R^1^, R^2^, and R^3^ positions while the BINOL-Cp scaffold itself is unchanged. Proceeding in this manner resulted in the generation of an additional 366 valid structures (out of 1,000 initial structures generated), many of which lie in the previously sparsely populated regions of the O1/O4 map (Figure 6c), showing success in broadening the steric diversity of the ligand set. Examination of the specific structures shows our diffusion model generates BINOL-Cp derivatives with R-groups that can either selectively block substrate approach from one side (O1 or O4) or, in extreme cases such as **3**, shield the metal center from nearly all directions. Such steric diversity allows for systematic evaluations of how steric hindrance in specific quadrants or octants influences the reaction outcome, providing a foundation for developing improved stereochemical models of asymmetric transformations that extend beyond “back-of-the-envelope.”

**Figure 6:**
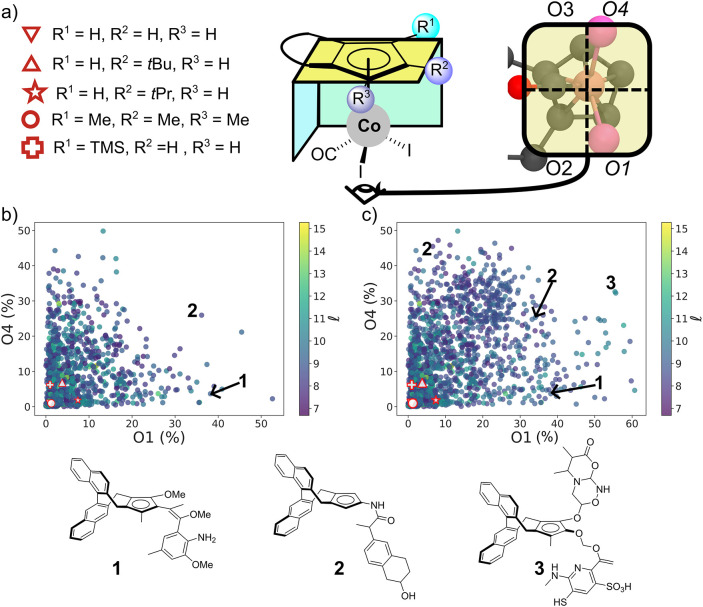
(a) Reference experimental BINOL-Cp ligands investigated in Ozols et al.^89^ and bottom view of the Co­(III) complex. O1/O4 steric property maps of generated Cp ligands obtained by (b) molecular outpainting alone and (c) molecular outpainting augmented with inpainting of the selected generated molecules.

To probe different steric environments of the back and side walls that occupy Q2 and Q3 we explored alternative backbones to BINOL. Starting from BINOL-Cp structures bearing hydrogen substituents at the R^1^, R^2^, and R^3^ positions, the inpainting method was applied to the BINOL backbone while keeping the Cp ring and hydrogen substituents fixed. Importantly, we restrict the model to produce new ligands that retain the molecular size as the BINOL-Cp. The extent of backbone modification during inpainting is controlled by the denoising strength parameter *d* in the molecular inpainting algorithm (Algorithm 2), with larger *d* values producing greater deviations from the initial structure. We evaluate the validity of the generated ligands using the same criteria described above and assess their steric properties following the same workflow. For each denoising strength (*d*), we generate 1,000 candidate structures. At low denoising strength (*d* = 0.3), the method yields the highest fraction of valid Cp ligands (87%), although most correspond to the duplicate of the input BINOL-Cp structure with only minor geometrical variations. As *d* increases, the generated ligands become more structurally diverse, spanning a broader region of the Q2/Q3 property map and including molecules distinct from BINOL-Cp (Figure 7a). However, this increase in diversity comes at the expense of a lower overall success rate, as higher denoising strengths more frequently produce invalid structures (Table S4). Figure 7b shows examples of the rich structural diversity in the different ligands created, ranging from those having considerable steric bulk in Q3 due to the presence of an indole moiety but a relatively open Q2 (**4**) to ligands such as **5** and **6** that are congested in both Q2 and Q3. In principle, ligands such as these would possess very effective “back wall sterics”, thereby permitting computational analysis, testing, and validation of the efficacy of the current heuristic “back-of-the-envelope” design models. Importantly, hypothetical structures such as these can serve a valuable role as conceptual probes for developing and refining ligand design models, even if many of the proposed ligands are not synthetically feasible.

**Figure 7:**
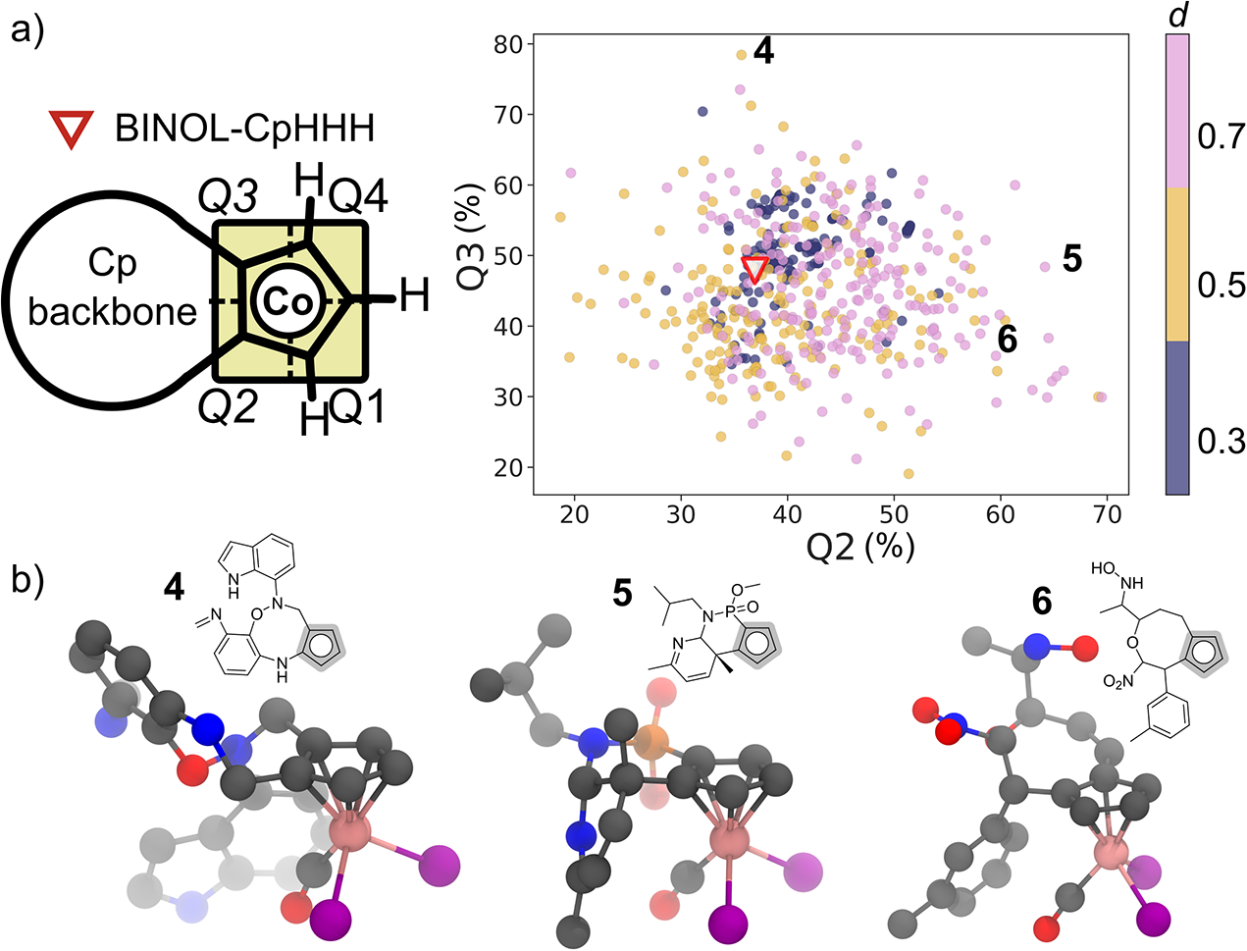
(a) Q2/Q3 steric property maps for Cp ligands generated via inpainting the BINOL backbone under varying denoising strengths. (b) Representative examples of generated asymmetric Cp generated with inpainting.

#### Designing Molecules with Target Geometrical Features

The next illustrative example is a molecular inverse design problem in which specific 3D geometrical features, such as targeted bond distances and relative orientations of functional groups, are the design objectives. For this case study, we consider the inverse design of intramolecular frustrated Lewis pairs (IFLPs) for catalytic hydrogenation of CO_2_ to formate (CHTF) (Figure 8a). IFLPs are bifunctional molecules in which a Lewis acid and a Lewis base are tethered within the same framework but sterically prevented from quenching each other, thereby creating reactive sites for small-molecule activation.^93–95^ In the catalytic cycle, the IFLP first activates H_2_, forming the **INT2** intermediate, where the Lewis acid binds H^–^ and the Lewis base binds H^+^. This intermediate then binds with CO_2_ (**INT3**) and subsequently forms formate (**INT4**), after which the catalyst is regenerated. Previous studies^32,96^ established two key factors governing IFLP catalytic performance: (i) the geometrical attributes of the **INT2** intermediate, specifically the B–N distance (*d*_*BN*_) and the angle between the B–H and N–H bonds (Φ), and (ii) the acidity and basicity of the Lewis centers. As a proof of concept for structure-directed generation, we focus on controlling the geometrical attributes of **INT2**, targeting *d*_*BN*_ values between 2.4–3.2 Å and Φ values between 70–140°, which correspond to high catalytic activity as established in previous studies^32^ (Figure 8b). This design task presents two key challenges: (i) the generated molecules must incorporate valid Lewis units that bind with hydride and proton to form chemically plausible **INT2** structures, and (ii) the *d*_BN_ and Φ of resulting **INT2** must lie within the target region of the property map.

**Figure 8:**
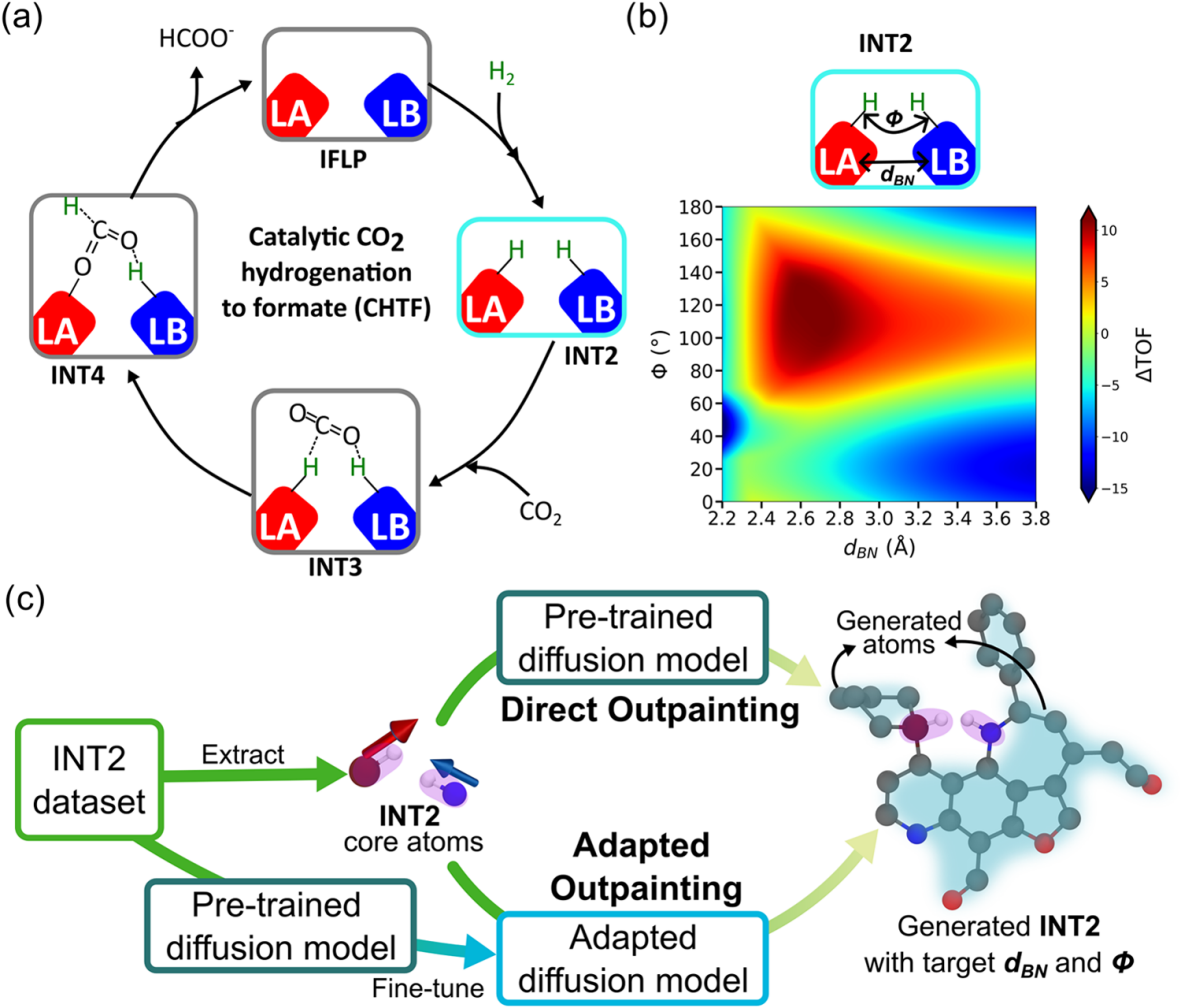
(a) Reaction mechanism of CHTF with the IFLP. (b) Activity map showing the relative turnover frequency (ΔTOF) for CHTF as a function of d_*BN*_ and Φ for B/N IFLPs. The ΔTOF is computed as log­(TOF/TOF_*uLP*_) where uLP is the unconstrained intermolecular B/N Lewis pair. *Adapted from ref* ^34^. *Available under a CC-BY 4.0 license. Copyright 2022 Author(s).* (c) Molecular outpainting methods over B–H and N–H motifs (highlighted in green) with target *d* and Φ values to generate **INT2** structures.

To tackle this molecular design problem, we employ the outpainting method, a structure-guided generation technique that extends existing molecular fragments by generating additional atoms around a fixed core structure, effectively completing the molecular scaffold during the diffusion process. This makes the outpainting approaches particularly suitable for inverse design applications where geometrical features are the primary design targets, as well as for scenarios requiring the preservation of specific atoms or functional motifs to ensure chemically relevant candidates.

As input for outpainting generation, we extract an **INT2** structure with the desired geometrical properties (*d*_*BN*_ = 2.82 Å, Φ = 94.8°) from the curated **INT2** data set derived from the CoRE MOF 2019 database (Figure 8c).^97^ The substructure, containing the B–H and N–H motifs, is used as the input scaffold, from which 1,000 candidate **INT2** structures are generated using this guidance method. A 3D structure is classified as a chemically valid **INT2** if it satisfies the following criteria:

1. All atoms satisfy standard valency rules.
2. The hydride and proton atoms in the core scaffold bond exclusively to the B and N Lewis centers, respectively.
3. The B and N centers belong to the same connected molecule.

While directly using the outpainting method with the pretrained diffusion model was successful to enrich the BINOL-Cp scaffold in the previous case study, its performance on the BH/NH scaffold of **INT2** is unsatisfactory, yielding only 5.2% valid **INT2** structures. This low success rate arises from the sparsity of the **INT2** core, which lacks the spatial rigidity provided by the Cp scaffold in the previous example. Incorporating an extended **INT2** scaffold that includes BH/NH motifs along with adjacent atoms around the Lewis centers (Figure S4; see SI for details) modestly improves the validity to 9.8%. Additional discussion of the distorted structures obtained in this setting is provided in the Supporting Information.

To better tailor the diffusion model for outpainting generation, we introduce the adaptive outpainting strategy, specifically developed to address the shortcomings of direct outpainting on sparse scaffolds such as the BH/NH core of **INT2**. This involves fine-tuning the pretrained diffusion model on the **INT2** dataset described above with a specialized molecular outpainting objective (Algorithm 4, Figure 8c) for 200 epochs. During fine-tuning, the core atoms of **INT2** (i.e., B–H and N–H motifs) are kept fixed, conditioning the model to generate complete **INT2** structures around these motifs. This training scheme specifically tailors the diffusion model to complete the structure from the BH/NH core, potentially offering improved performance in the case where the direct outpainting method fails due to limited spatial and chemical constraints. With the adapted diffusion model, we follow the generation and evaluation procedure as described above.

Using the same BH/NH scaffold of **INT2** as in direct outpainting, the adaptive approach yields a substantially higher fraction of chemically valid and connected **INT2** structures (38.1%). However, this scaffold, while possessing the desired *d*_*BN*_ and Φ values, is underrepresented in the data set, making the performance of adaptive outpainting sensitive to the choice of input scaffold. When employing a more common BH/NH scaffold (*d*_*BN*_ = 2.67 Å and Φ = 33.45°) instead, the success rate further improves, yielding 46.7% valid **INT2** structures.

Next, we evaluate the generated **INT2** structures after geometry relaxation at the GFN2-xTB level, since the relevant properties must be assessed at their equilibrium geometries. At this stage, a valid **INT2** must not only remain chemically intact but also preserve the original topology of the core scaffold upon optimization. The key geometrical descriptors, *d*_*BN*_ and Φ, are then remeasured from the optimized geometries to assess whether they remain within the desired region of the property map. We define postoptimization validity as an optimized **INT2** structure that satisfies the chemical validity criteria outlined above, and a postoptimization hit as one that additionally retains *d*_*BN*_ and Φ within this target region. We observe a greater fraction of valid **INT2** molecules after geometry relaxation, as the optimization step can correct distortions and enforce chemically consistent structures. However, in some cases relaxation disrupts the core scaffold, leading to loss of the intended **INT2** topology. A higher proportion of structures generated with the common BH/NH scaffold remain chemically intact after optimization (83.8%) compared to those generated with the target BH/NH scaffold (64.1%). Yet, fewer of the optimized structures from the common scaffold fall within the target region of *d*_*BN*_ and Φ­(18.6% vs 25.7%, Table S5).

Akin to the previous work,^96^ most of the generated IFLPs with favorable d_*BN*_ and Φ values possess two or three atoms between the B and N centers (Figure 9b), corresponding to cis-vicinal or ansa-type arrangements. Representative **INT2** of IFLP candidates with the target distances and angles are shown in Figure 9c. Despite a non-negligible number of structures satisfying the desired geometrical criteria, the hit rate after geometry optimization could be improved through active learning, where valid and hit **INT2** structures are iteratively added to the training set to refine the model. The generated IFLPs also show higher average SCScore^98^ and SAScore^99^ than those in the training data set, suggesting room to improve chemical relevance and synthesizability. Notably, some of the generated molecules contain structurally exotic motifs (Figure 9c), such as quinoidal patterns and polynitrogen heteroaromatic systems. Additionally, chemical constraints such as the acidity and basicity of the Lewis centers^100^ are not considered in the present guidance diffusion framework, primarily due to the difficulty of generalizing such structure–property relationships required to guide generation based on these chemical properties.

**Figure 9:**
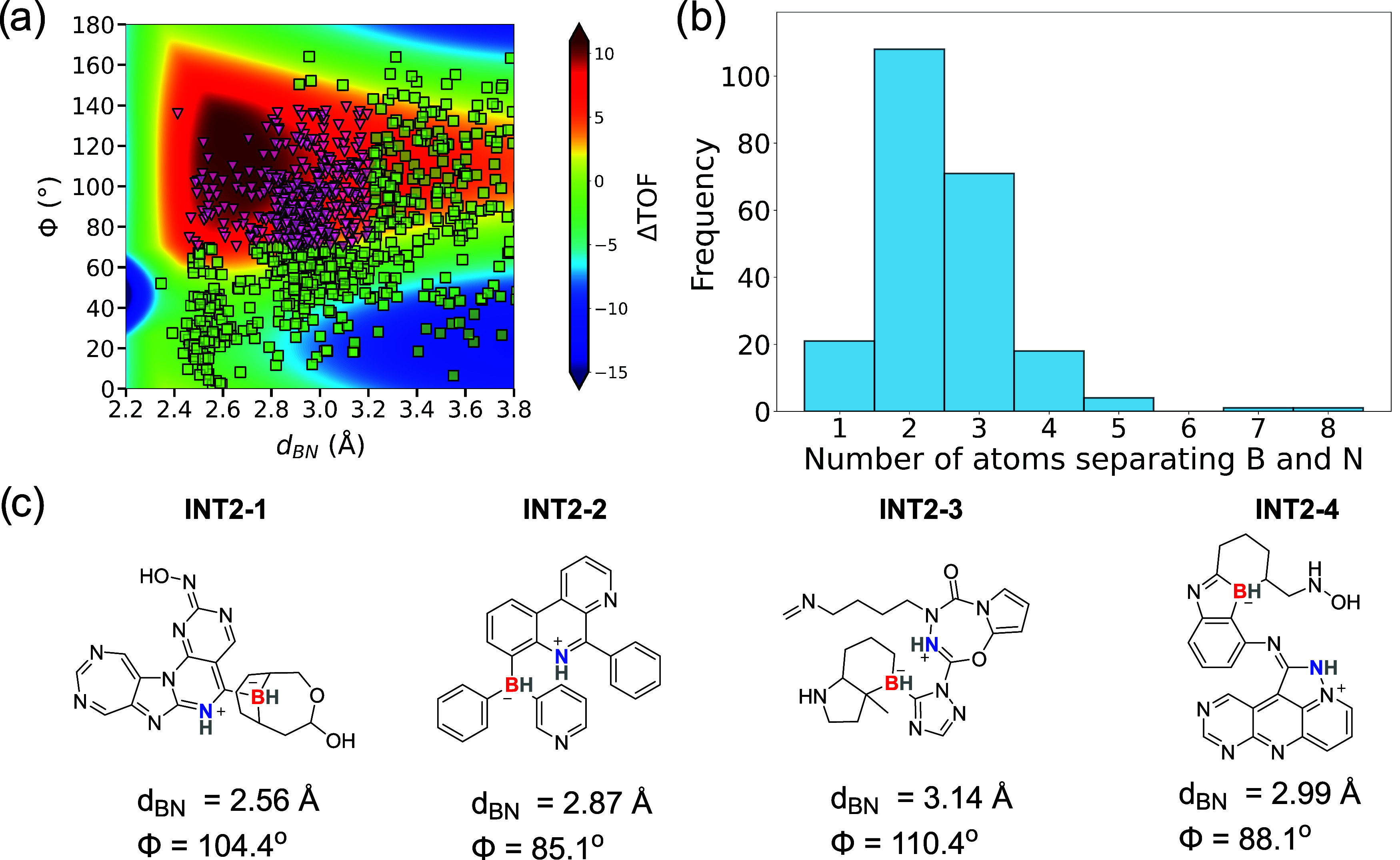
(a) Activity map showing the relative turnover frequency (ΔTOF) for CHTF as a function of d_*BN*_ and Φ for B/N IFLPs. The ΔTOF is computed as log­(TOF/TOF_*uLP*_) where uLP is the unconstrained intermolecular B/N Lewis pair considered in the previous work.^32^ for B/N IFLPs; each point corresponds to a generated hit IFLP molecule optimized at the GFN2-xTB level. (b) Distribution of the number of atoms separating the Lewis centers. *Adapted from ref* ^34^. *Available under a CC-BY 4.0 license. Copyright 2022 Author(s).* (c) Representative examples of generated **INT2** candidates.

To explore potential improvements, we evaluated an augmented diffusion model incorporating additional atomistic features in the molecular representation. While this model generates a higher fraction of valid FLP candidates prior to geometry optimization compared to the original model, the proportion of postoptimization valid and hit FLP molecules remains lower (Table S4). Representative FLP molecules generated by these EDM variants are shown in Figure S8. These results call for the development of more robust 3D generative models and tailored training and guidance strategies capable of more effectively constraining the generative process toward chemically consistent structures.

#### Designing Molecules with Target Molecular Properties

Apart from guiding the generative process toward specific structural features, the pretrained diffusion model can also be adapted for inverse molecular design by steering sampling toward target molecular characteristics computed from or predicted for a 3D structure (e.g., vertical excitation energies and ionization energies). To enable this, we employ three guided diffusion techniques: GG, CFG, and a hybrid method combining both methods (presented in the Method section). Here, we want to demonstrate the ability of our framework to generate molecules with target properties and assess the performance of these three guidance methods. As a case study, we revisit the problem of discovering singlet fission (SF) chromophores—a molecular design problem we addressed previously using different generative approaches, including: a genetic algorithm,^12,59^ and a smiles-based generative model.^101^ These organic compounds are of particular interest due to their potential to enhance solar energy conversion by generating two triplet excitons from a single absorbed photon.^102–105^

For molecules to be considered viable SF chromophores,^102–105^ they must satisfy vertical excited-state criteria: Near-thermoneutral singlet fission driving energy (Δ_SF_ ≡ *E*_S_1_,ve_ −2*E*_T_1_,ve_ ≳ −1 eV); emission in the absorption range of silicon (*E*_T_1_,ve_ > 1.1 eV); and absorption of abundant photons in the solar spectrum (*E*_S_1_,ve_ < 3.8 eV), where *E*_S_1_,ve_ and *E*_T_1_,ve_ are the vertical excitations to S_1_ and T_1_ at the ground-state geometry. Importantly, generated candidates must satisfy these criteria after ground-state geometry optimization. This adds complexity to the molecular design problem, as the generative model must produce molecules that not only possess reasonable ground-state geometries but also exhibit the desired excited-state properties when subjected to quantum chemical evaluation. In what follows, we define the energy score function, as formulated in previous work,^106^ to quantify the suitability of a molecule for SF applications (more details in the Method section).

Regardless of the guidance approach considered (CFG, GG, or hybrid), we need a data set of molecules with computed S_1_ and T_1_ energies to train property prediction models and define target criteria. For this purpose, we leverage the FORMED database,^107^ a data set of 117k experimentally reported organic crystal structures with computed ground- and excited-state properties at the DFT level. The excited-state properties in FORMED were computed using TD-DFT at ωB97*X*/6-31G* level, providing the reference data needed for our SF application.

To enable rapid evaluation of the generated molecules, we train an EGNN-based surrogate model to predict *E*_S_1_,ve_ and *E*_T_1_,ve_ directly from 3D molecular structures. This predictive model, trained on the FORMED database using the same TD-DFT reference values (ωB97*X*/6-31G*), serves as the evaluation function for both gradient-based guidance and initial candidate screening. Final validation of promising candidates is performed using full xTB geometry optimization followed by TD-DFT.

The three guidance methods explored here require different training strategies (Figure 10a). In what follows, we briefly summarize the training procedure for each guidance method:

1. Gradient Guidance (GG): This method uses the gradient of the evaluation objective to guide the denoising process. Accordingly, we need to evaluate the gradient of the objective at every denoising step, meaning the surrogate model must compute the gradient of the target function with respect to the structures generated at each step (including noisy representations). We therefore train an EGNN-based model to predict the target function established above from diffusion-perturbed inputs. Because this depends on the diffusion model’s noise schedule and parametrization, the surrogate must be trained specifically for each diffusion model and retrained if the architecture changes. For GG, we define a guidance scale and a gradient-clipping threshold to prevent excessive deviation from the pretrained sampling trajectory.
2. Classifier-Free Guidance (CFG): This method relies on a conditioned generative model to guide the diffusion process. To achieve this, we need a diffusion model conditioned on the target properties. This requires fine-tuning the pretrained diffusion model on the FORMED dataset with the target function as the condition. To incorporate property conditions into the EGNN, we extend the input dimension of the EGNN encoder layer and initialize the additional channels randomly. For the CFG approach, we define the target value for the property to optimize and the guidance scale. In this case, we maximize the target function defined above. Property values are scaled by a factor of 0.5, with a dropout ratio of 0.2 applied to the conditions and null values set to −4. CFG can be used with multiple target values to generate molecules that satisfy multiple objectives (this is discussed in the Supporting Information).
3. Hybrid Guidance (CFG/GG): This approach combines the strengths of both GG and CFG. The two guidance approaches can be used together in a hybrid mode, where the conditioned diffusion model is used in parallel with the gradient of the target function to guide the diffusion model. For the hybrid approach, we define the guidance scale for both CFG and GG, as well as the number (or fraction) of denoising steps where gradient guidance is applied.

**Figure 10:**
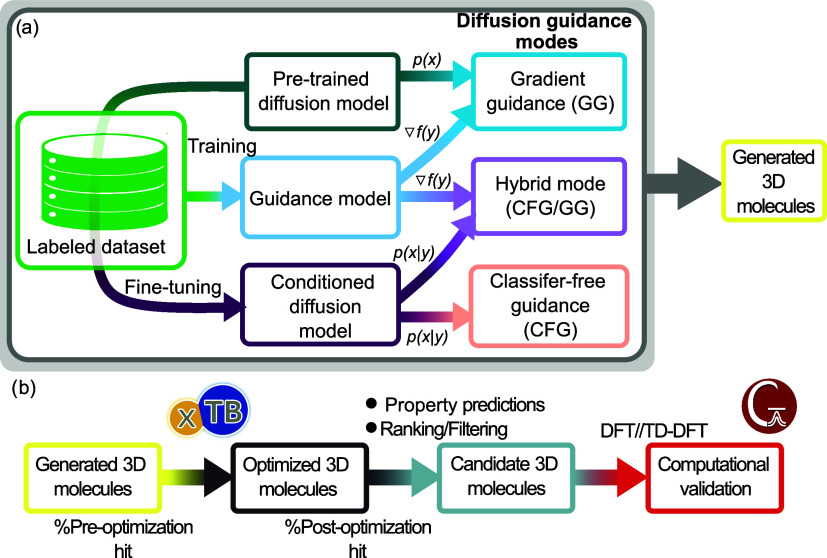
a) Workflow of the property-directed molecular generation leveraging a pretrained diffusion model to build guidance models and classifier-free guidance models. These models are then used to steer the denoising process to 3D molecules satisfying target properties. b) Workflow for selecting candidate 3D molecules for computational validation.

To compare the performance of these three guidance approaches, we define a set of hyperparameters for each method (with details and sensitivity in the Supporting Information) and focus in the main text on representative settings. We generate 1000 molecules with each guidance mode and assess performance by comparing the resulting distributions of energy scores of the generated molecules against those from the pretrained, unguided model (Figure 11a). All three methods shift the distribution of the energy score toward more positive values relative to the FORMED database and the unguided model. CFG alone struggles to push the energy score near the edge of the FORMED distribution, likely due to the limited representation of SF-satisfying molecules in FORMED, which constrains the conditional signal. In contrast, GG effectively shifts the distribution toward positive values, with many molecules achieving an energy score value higher than 0. The hybrid approach leverages both effects: CFG biases sampling toward the desired property manifold, while GG fine-tunes trajectories via gradients, yielding the largest positive shift.

**Figure 11:**
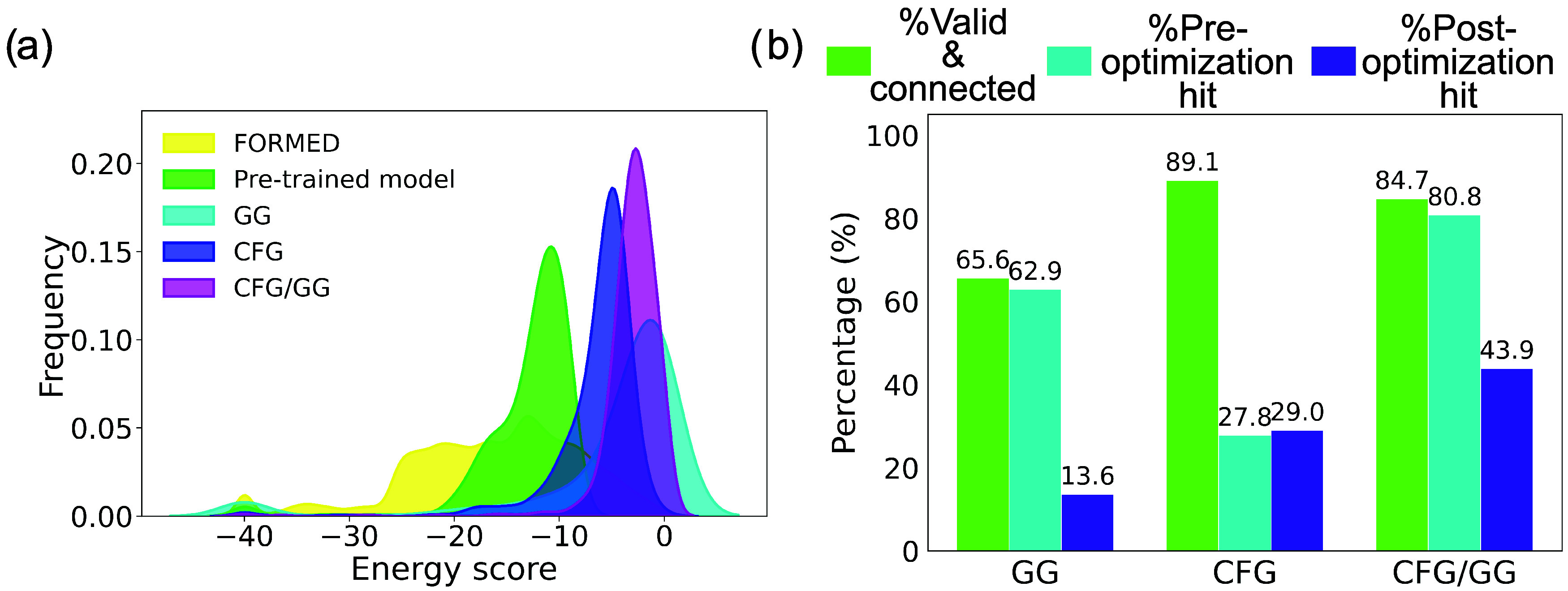
(a) Kernel density histogram plots of the energy score value predicted with the regressor model for 1000 molecules generated using a pretrained diffusion model, GG, CFG, and CFG/GG guidance modes, compared against molecules from the FORMED database. (b) The bar plot summarizes the performance of GG, CFG, and CFG/GG. The percentage values are calculated with respect to the total number of molecules generated.

Apart from shifting the distribution of the target function, we need to assess the ability of the guidance approaches to generate molecules that are chemically valid. Here, we define chemical validity as the ability of the generated molecules to pass basic chemical checks, including valency checks, the absence of disconnected fragments, and the presence of chemically reasonable bonding patterns (as defined in the Method section and Supporting Information). The results for the three methods are shown in Figure 11 and Table S8. We find that the CFG approach achieves the highest chemical validity (89.1%), followed by CFG/GG (84.7%) and GG (65.5%). Both CFG and CFG/GG have a similar validity value as the pretrained model. The use of the gradient to guide the generation of the GG lowers the chemical validity of the generated molecules. Clamping the gradient or using a lower guidance strength increases the validity of the molecules at the expense of the target function optimization (Table S7). For CFG, increasing the CFG scale or changing the target value does not improve the hit rate but considerably reduces the validity of the generated molecules (Table S6).

To further assess the performance of the three guidance methods, we perform geometry optimization on the generated molecules using GFN2-xTB and predict the properties of the molecules using the new geometries. This step is crucial, as the properties of interest are computed at the ground-state equilibrium geometry, and the generation process does not guarantee that the generated structures are at a local minimum. We define postoptimization hits as molecules that satisfy the SF criteria after geometry optimization and property prediction. In the same spirit, we define preoptimization hits as the molecules that satisfy the SF criteria before optimization. The postoptimization hits discard the molecules that are not valid prior to the optimization step. The results are shown in Figure 11b. We find that the hybrid approach achieves the highest postoptimization hit rate (43.9%), followed by CFG (27.8%) and GG (13.6%). Although GG achieves a high preoptimization hit rate (62.9%), the majority of these hits are not chemically valid. The introduction of gradient into the generative process distorts the geometries (the average RMSD is 1.02 Å, compared to the optimized geometries), forcing structures to exhibit target properties at the cost of the quality of their 3D structure (Figure S11). CFG, on the other hand, achieves a lower preoptimization hit rate (27.8%) but retains most of these hits after geometry optimization. Interestingly, in some cases, molecules that initially failed to meet the SF criteria become valid postoptimization hits (Table S6). The hybrid approach effectively combines the strengths of both methods, achieving a high preoptimization hit rate (80.8%) and retaining a significant portion of these hits after geometry optimization. Both CFG and the hybrid approach produce high-quality 3D molecular structures, with average RMSD values of 0.53 and 0.57 Å, respectively, compared to their optimized counterparts. We discuss these results in more detail in the Supporting Information section 8. Considering both 3D structure quality and guidance effectiveness, the hybrid method demonstrates the best overall performance, offering superior sampling efficiency and a higher likelihood of generating viable SF chromophores compared to CFG (27.8%) and GG (13.6%). The hybrid approach effectively combines the strengths of both methods, leveraging the ability of CFG to generate molecules with desired properties and the fine-tuning capability of GG using the gradient of the target function. This results in a more efficient exploration of chemical space and a higher likelihood of generating viable SF chromophores. We further note that SF candidates generated using these property-directed guidance methods alone tend to exhibit relatively high synthetic complexity, with average SCScores of approximately 3.9 (Tables S6 and S7). Incorporating SCScore as an additional conditioning variable during guidance generation provides a mechanism to bias the model toward chemically more feasible structures, leading to a lower average SCScore for the generated molecules (∼3.4). However, this improvement comes with a slight reduction in hit rate (Table S6), reflecting a trade-off between optimizing target excited-state properties and enforcing feasibility-related criteria.

On top of the performance metrics discussed above, we also assess the diversity and novelty of the generated molecules. For the generative models, this is an important aspect to consider, as we want to ensure that the models are not simply memorizing the training data but are able to generate new and diverse molecules. We compute the uniqueness and novelty of the postoptimization hits generated by the three guidance methods. Uniqueness is defined as the dissimilarity between the molecules generated using Tanimoto similarity metrics and Morgan fingerprint representation. Novelty is defined as the average distance between the molecules generated and those in the data set that satisfy the SF criteria. The results are shown in Table S8. We find that all three guidance methods achieve high uniqueness and novelty scores, ranging from 0.70 to 0.80 for uniqueness and from 0.65 to 0.70 for novelty. For comparison, the molecules in the FORMED data set that satisfy the SF criteria have a uniqueness of 0.49. In comparison, the RL-based approach^12,59,108–110^ optimizes molecular generation policies to achieve higher hit rates, but the resulting molecules are often structurally similar and concentrated within narrow regions of chemical space in each trial.^101^ As a result, external structural constraints and multiple optimization runs are required to ensure broader exploration.

In what follows, we focus our subsequent analysis on the chemical structures generated and the computational validation of the postoptimization hits generated using the hybrid guidance method. We consider here the top 150 molecules according to their postoptimization SF score. This following analysis aims to discuss the potential of the diffusion model to generate novel, diverse, and chemically relevant SF chromophores. Among the 150 molecules, 134 molecules satisfy the vertical SF criteria. We also consider a more stringent condition based on adiabatic excited-state energies, requiring that viable SF chromophores satisfy S_1,ad_ – 2T_1,ad_ ≥ 0 eV. Based on this criterion, we identify 69 molecules as viable SF chromophores. Thanks to our diffusion model’s ability to explore chemical space, we discover a diverse range of SF chromophore families.

Examining the illustrative examples of molecules generated by the EDM in Figure 12, we observe a notable prevalence of heterocyclic compounds, including fulvenoid and azoxy moieties, features consistent with previous studies.^101^ Alongside these desirable motifs, the model also generates molecules with less favorable or potentially artifactual chemical characteristics that are not penalized by standard synthesizability scores, such as SCScore and SAScore. Noteworthy issues include reversed π-conjugation in azo switches (**4**), free boron centers susceptible to hydrolysis (**11**) and synthetically challenging heterocycles (**1**, **10** ). While these examples highlight current limitations of the proposed candidate molecules, they also indicate clear opportunities for improvement. MolCraftDiffusion is designed as a modular framework within which alternative 3D generative models and more robust guidance mechanisms can be readily implemented to improve generation quality and hit rates. Motivated by the successful incorporation of SCScore into EDM trained on the GEOM data set, which results in the generation of molecules with lower average SCScore values compared to unconditional generation (Figure S3), we further explore this capability by introducing SCScore as an additional conditioning signal alongside the SF energy targets. In parallel, we augment the molecular representation by incorporating additional atomistic features into the diffusion model.

**Figure 12:**
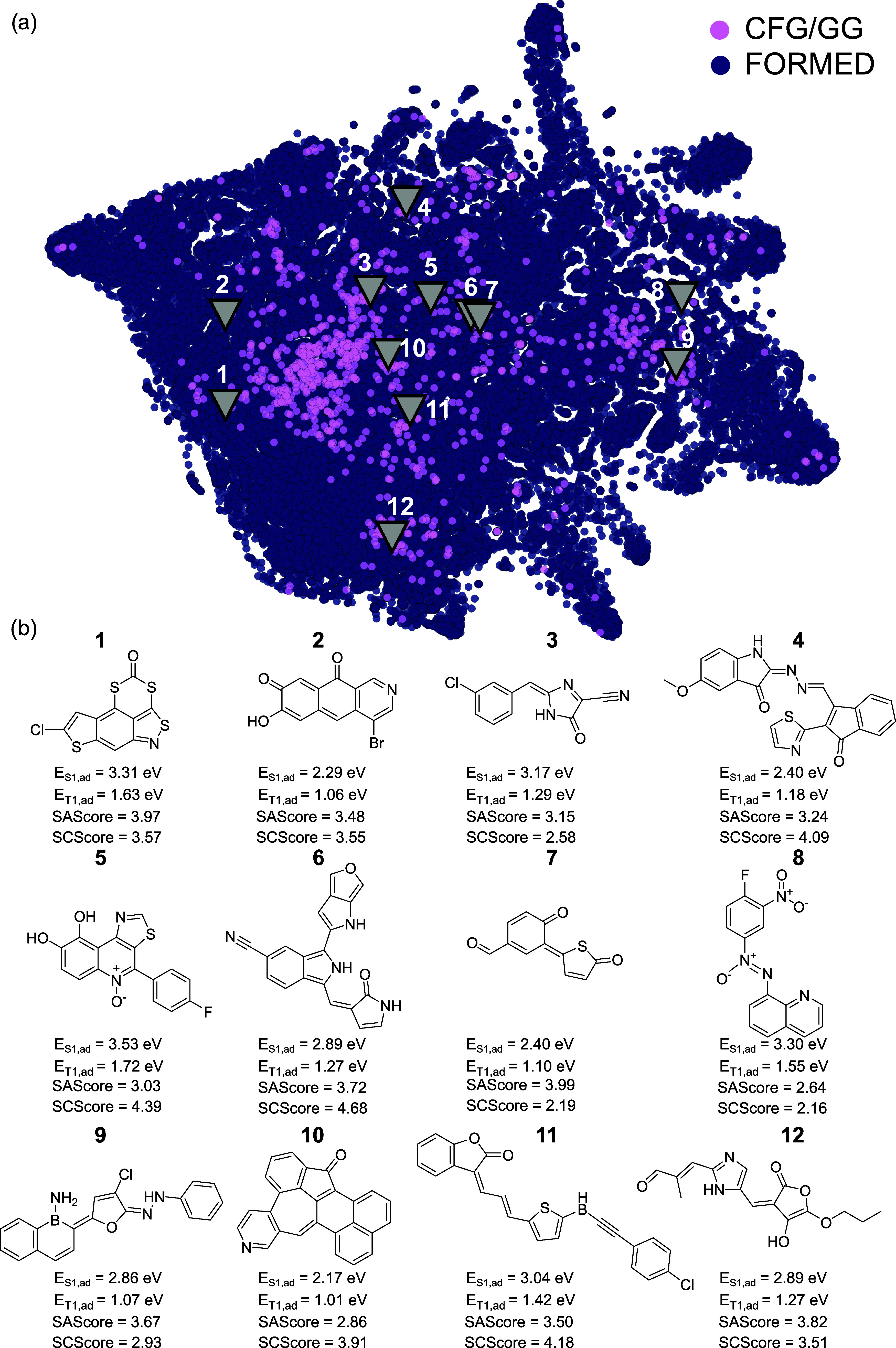
(a) t-SNE plot generated from the Morgan fingerprint representation of the predicted SF chromophores generated by the diffusion model guided by the hybrid guidance and molecules in the FORMED data set. (b) Selected candidate molecules generated by the diffusion model and their computed adiabatic S_1_ and T_1_ energies with TD-DFT (ωB97X-D/6–31G­(d)), as well as their SAScore and SCScore values.

Under otherwise identical settings, this modification leads to improved structural quality of the generated molecules, as reflected by a higher proportion of chemically valid and connected molecules and reduced distortion relative to optimized geometries (Table S6). In addition, the generated molecules exhibit lower average SCScore values (3.4) compared to those created by the original EDM (3.9). Representative molecules generated using the original EDM and the augmented model incorporating additional atomistic features and SCScore conditioning are shown in Figure S13. Compared to molecules generated with the original model, those produced by the augmented model exhibit no third-row elements and fewer chemically unstable or reactive functional groups, such as ketenes, hydroxyhydrazines, and Se–S motifs, suggesting that SCScore conditioning helps constrain the generation toward more chemically feasible target molecules.

### Extensibility of the Platform

Having demonstrated the versatility of MolCraftDiffusion across a range of molecular generation tasks using our preliminary diffusion model (EDM), we now illustrate the modular nature of the platform by porting additional 3D generative architectures from the literature. Crucially, integrating these external models required no modifications to the core codebase.

As general-purpose unconditional generators, we integrated TABASCO,^66^ a Transformer-based atomistic diffusion model operating under a flow-matching framework, and ADiT,^48^ an all-atom latent diffusion transformer. Both models were trained on the GEOM data set for 600,000 steps and evaluated using the same analysis pipeline described above. Results are reported in Table S3 and show competitive performance relative to our EDM trained with HCL and to models from Nikitin et al.^111^ Because all models (EDM-HCL, TABASCO, and ADiT in Table S3) share the same training, generation, and evaluation protocols, MolCraftDiffusion can serve as a unified benchmarking platform for comparing the generative capabilities of diverse molecular diffusion architectures, as more 3D generative models will be included in the future.

To demonstrate extensibility to specialized generative tasks, we integrated ShEPhERD^67^ into our MolCraftDiffusion, an SE(3)-equivariant diffusion model for pharmacophore-conditioned generation that jointly operates on 3D molecular structures and their interaction profiles, encompassing molecular shape, electrostatic potential surfaces, and directional pharmacophores. Alongside the model, we integrated the ShEPhERD scoring functions, a suite of metrics for evaluating 3D molecules based on their interaction profile, into our evaluation and analysis module. To verify the implementation, we loaded the ShEPhERD-GDB17 pretrained weights from the original work and assessed self-consistency between the generated interaction profiles and those computed directly from the generated 3D structures. Consistent with the results reported by Adams et al.,^67^ generated shape and ESP profiles show high self-consistency, closely matching their true profiles (Figure S15). In contrast, pharmacophore self-similarity is lower than that observed for shape and ESP, although it remains higher than the similarity to randomly selected molecules from the ShEPhERD-GDB17 data set.

## Conclusion

We have introduced MolCraftDiffusion, a modular and extensible platform for building, deploying, and evaluating 3D molecular diffusion models in computational chemistry. The platform decouples core training logic from model definitions and task implementations, providing a consistent environment in which diverse generative architectures and guidance strategies operate under identical protocols. All workflows are accessible via a command-line interface with YAML configuration files, lowering the barrier to adoption for domain chemists. The extensibility of the platform is demonstrated by incorporating three architecturally distinct models from the literature, TABASCO, ADiT, and ShEPhERD, on top of the baseline EDM, without modifying the core codebase.

Within this platform, we implement curriculum learning for training and fine-tuning diffusion models, and integrate a suite of guidance mechanisms categorized by target attribute type. Structure-directed methods include molecular inpainting, for systematic exploration of structural variants around a reference molecule, and outpainting, for completing 3D structures conditioned on user-defined scaffolds. Property-directed generation is supported via gradient-based and classifier-free guidance, steering the denoising process toward molecules satisfying target physicochemical criteria.

We demonstrate these control generation capabilities across several downstream tasks. First, we construct a virtual library of asymmetric cyclopentadienyl ligands based on the BINOL-Cp scaffold, systematically varying structures to explore a broad range of steric profiles. In addition, we apply MolCraftDiffusion to molecular inverse design: generating intramolecular frustrated Lewis pairs satisfying target geometric criteria and singlet fission chromophores meeting excited-state criteria. In the singlet fission application, we observe that the quality of generated 3D molecular structures can be improved under the same generative model through richer molecular representation with additional atomistic features, whereas results for the IFLP system, involving larger molecular size remain more challenging and less conclusive under the same enhancements.

More broadly, MolCraftDiffusion provides a common foundation for integrating, comparing, and extending current and future advances in 3D molecular generative modeling, supporting continued methodological progress in data-driven computational chemistry.

## Supplementary Material



## Data Availability

The MolCraftDiffusion software package is available publicly at https://github.com/lcmd-epfl/MolCraftDiffusion. The trained models and a compiled data set can be found at https://huggingface.co/pregH/MolecularDiffusion. The executable YAML configuration files corresponding to each experiment described in the manuscript are provided at https://zenodo.org/records/19511401. Tutorials covering the configuration of the YAML files for all workflows are available publicly at https://preghosh.github.io/MolCraftDiffusion/.

## Notes

Detailed description of the diffusion model and training procedures; composition and statistics of the compiled 3D molecular data sets; additional implementation details and hyperparameters; definitions of structure quality and PoseBusters metrics; supplementary results for curriculum learning, structure-guided virtual library generation, and inverse molecular design applications; parameter sensitivity analyses for guidance methods; additional figures and tables supporting the main text; ablation study of diffusion models; software implementation details (PDF)
