# Supplementary material for: Modular Framework for 3D Molecular Generation in Computational Chemistry Applications
Source: J Am Chem Soc. 2026 Jun 22;148(25):25370–88. doi: 10.1021/jacs.5c19960 (PMC13339657; doi:10.1021/jacs.5c19960)
Supplement: Supplementary file 1 [file ja5c19960_si_001.pdf]

A Modular Framework for 3D Molecular Generation in Computational Chemistry Applications

Thanapat Worakul,† Mohammed Azzouzi,‡ Matthew D. Wodrich,†,¶ and Clémence Corminboeuf*,†,¶

†*Laboratory for Computational Molecular Design, Institute of Chemical Sciences and Engineering, Ecole Polytechnique Fedéralé de Lausanne (EPFL), 1015 Lausanne, Switzerland*

‡*Laboratory for Computational Molecular Design, Institute of Chemical Sciences and Engineering,*

École Polytechnique Fédérale de Lausanne (EPFL), 1015 Lausanne, Switzerland

¶*National Center for Competence in Research-Catalysis (NCCR-Catalysis), École Polytechnique Fédérale de Lausanne (EPFL), 1015 Lausanne, Switzerland*

E-mail: clemence.corminboeuf@epfl.ch

Table of Contents

S1: Diffusion Model for 3D Molecule Generation **3**

S2: 3D Chemical Structure Database **7**

S3: Structure Quality Metrics **9**

S4: Implementation Details **11**

S5: Additional Results for Unconditional Generation with Diffusion Models **14**

S6: Additional Results for Virtual Library Design **15**

S7: Additional Information for Designing Molecules with Target Geometries via Structural Guidance Diffusion **16**

S8: Additional Information for Designing Molecules with Target Molecular Properties via Guidance Diffusion **21**

S9: Ablation Study of Diffusion Model. **28**

S10: Pharmocophore-conditioned Generation **30**

S11: Software Implementation Details **31**

S1: Diffusion Model for 3D Molecule Generation

Molecular Representation

Here, we represent 3D molecules as a point cloud, $\boldsymbol{x} = (\mathbf{x}, \mathbf{h})$, where $\mathbf{X} = (\mathbf{x}_1, \dots, \mathbf{x}_N)$ and $\mathbf{h} = (\mathbf{h}_1, \dots, \mathbf{h}_N)$. Each $\mathbf{x}_i \in \mathbb{R}^3$ is the Cartesian coordinate of atom i, and each $\mathbf{h}_i \in \mathbb{R}^{n_f}$ is the atomistic feature consisting of the one-hot encoding of the atom type and atomic number. Similar to Hoogeboom's work,[1] we assume a fully connected graph, considering all interatomic interactions.

Diffusion Model

Diffusion models are a class of probabilistic generative models that transform complex data distributions into simple, tractable ones, typically Gaussian distributions. This framework comprises two key processes: (1) the forward diffusion process, which gradually adds noise to the data, systematically corrupting it until it resembles pure noise, and (2) the reverse diffusion process, which removes the noise to reconstruct the original data from a noisy input.

The forward diffusion process is defined by a multivariate normal distribution for all time steps t, transforming clean data $\boldsymbol{x}$ into a latent variable $\mathbf{z}_t$ at t time step

$$q(\mathbf{z}_t|\boldsymbol{x}) = \mathcal{N}(\mathbf{z}_t|\alpha_t \boldsymbol{x}, \sigma_t^2 \mathbf{I}) \tag{1}$$

Here α_t and σ_t determine the amount of signal retained and noise added, respectively, at the time step $t \in [0, 1]$. Both α_t and σ_t are determined from the noise schedule, which dictates the evolution of the noise level over time. Same as the implementation by Hoogeboom *et al.*,[1] a variance-preserving noise scheduler was employed,[2] ensuring that a constant total variance is maintained $\alpha^2 + \sigma_t^2 = 1$ at every time step.

With parameterization, $\mathbf{z}_t$ can be expressed as a linear combination of the original data $\boldsymbol{x}$ and noise variable ϵ

$$\mathbf{z}_t = \alpha_t \boldsymbol{x} + \sigma_t \epsilon, \text{where } \epsilon \sim \mathcal{N}(0, \mathbf{I}) \tag{2}$$

Given the Markov property of this process, the state at time t can also be derived from a previous state at time s (where $t > s$), the forward diffusion process can be written as:

$$q(\mathbf{z}_t|\mathbf{z}_s) = \mathcal{N}(\mathbf{z}_t|\alpha_{t|s}\mathbf{z}_s, \sigma^2_{t|s}\mathbf{I}) \tag{3}$$

where $\alpha_{t|s} = \alpha_t/\alpha_s$ and $\sigma^2_{t|s} = \sigma^2_t - \alpha^2_{t|s}\sigma_s$

The reverse diffusion process serves as a generative mechanism. It synthesizes new data by starting from a random noise sample $\mathbf{z}_T \sim \mathcal{N}(0, \mathbf{I})$ and progressively refining it through a series of denoising steps until it reaches $\mathbf{z}_0$, a sample resembling the original data distribution.

During this process, the clean data $\boldsymbol{x}$ is unknown. It is approximated by a denoising network $\phi(\mathbf{z_t}, t)$, which is trained to predict the noise $\hat{\epsilon}$, that was added to the data at time step t. From this prediction, an estimate of the clean data $\hat{\boldsymbol{x}}$, can be derived:

$$\hat{\boldsymbol{x}}_0 = \frac{1}{\alpha_t}(\mathbf{z}_t - \sigma_t \hat{\boldsymbol{\epsilon}}) = \frac{1}{\alpha_t}(\mathbf{z}_t - \sigma_t \phi(\mathbf{z}_t, t)) \tag{4}$$

The denoising step from time t to a previous time s is then performed as follows:

$$p(\mathbf{z}_s|\mathbf{z}_t) = \mathcal{N}(\mathbf{z}_s|\mu_{t\to s}(\hat{\boldsymbol{x}}, \mathbf{z_t}), \sigma^2_{t\to s,\mathbf{z_t}}\mathbf{I}) \tag{5}$$

where $\mu_{t\to s}$ and $\sigma_{t\to s}$ are defined as

$$\mu_{t\to s}(x, \mathbf{z}_t) = \frac{\alpha_{t|s}\sigma^2_s}{\sigma^2_t}\mathbf{z}_t + \frac{\alpha_s \sigma^2_{t|s}}{\sigma^2_t}x,$$
$$\sigma_{t\to s} = \frac{\sigma_{t|s}\sigma_s}{\sigma_t}.$$

The denoising network ϕ is trained by minimizing a weighted mean squared error (MSE)

between the predicted noise and the true noise:

$$\mathcal{L}(\boldsymbol{x}) = \mathbb{E}_{t\sim\mathcal{U}(0,1),\boldsymbol{\epsilon}\sim\mathcal{N}(0,\mathbf{I})}\left[w(\lambda_t)\cdot\frac{\mathrm{d}\lambda}{\mathrm{d}t}\cdot\|\hat{\boldsymbol{\epsilon}}-\boldsymbol{\epsilon}\|_2^2\right], \tag{6}$$

In this work, the product $w(\lambda_t)\cdot\frac{\mathrm{d}\lambda}{\mathrm{d}t}$ is simplified as $\frac{1}{2}(1-\texttt{SNR}(t-1)/\texttt{SNR}(t)$ where SNR represents the signal-to-noise ratio, defined as $\texttt{SNR}(t)=\alpha^2/\sigma^2$.

Once trained, the denoising generative process follows Algorithm 1

Algorithm 1 Unconditional 3D generation with the diffusion model

Input: Neural network ϕ, total steps T
Sample $\mathbf{z}_T \sim \mathcal{N}(0, I)$ ▷ Sample latent noise for atoms
Subtract the center of gravity of $\mathbf{z}_T^{(x)}$ in $\mathbf{z}_T = [\mathbf{z}_T^{(x)}, \mathbf{z}_T^{(h)}]$
for $t = T, \ldots, 0$ where s = t-1 **do**
  $\mu = \frac{1}{\alpha_{t|s}}\mathbf{z}_t - \frac{\sigma_{t|s}^2}{\alpha_{t|s}\sigma_t}\phi(\mathbf{z}_t, t)$
  $\sigma = \sigma_{t|s}\frac{\sigma_s}{\sigma_t}$
  Sample $\mathbf{z}_s \sim \mathcal{N}(\mu, \sigma)$
  Subtract the center of gravity of $\mathbf{z}_s^{(x)}$ in $\mathbf{z}_s = [\mathbf{z}_s^{(x)}, \mathbf{z}_s^{(h)}]$
end for
$[\mathbf{x}, \mathbf{h}] \sim p([\mathbf{x}, \mathbf{h}]|\mathbf{z}_0)$
Output: Generated molecule $[\mathbf{x}, \mathbf{h}]$

Equivariant Graph Neural Network

In our diffusion model, we employ the Equivariant Graph Neural Network (EGNN) as the denoising network $\phi(\mathbf{z}_t, t)$.[1] The EGNN is designed to respect the inherent symmetries of 3D molecular structures. This property, known as E(3) equivariance, ensures that any rotation, reflection, or translation applied to the input molecule results in an equivalent transformation of the output. Formally, for any orthogonal matrix Q and translation vector g, the network satisfies:

$$Q\mathbf{x}^{l+1} + g, \mathbf{h}^{l+1} = \mathbf{EGNN}(Q\mathbf{x}^l + g, \mathbf{h}^l) \tag{7}$$

The EGNN architecture is composed of a stack of Equivariant Graph Convolutional

Layers (EGCL). Each layer updates the node features and coordinates through a series of message-passing operations. The EGNN serves as the denoising function of the diffusion model, predicting the noise added to both the coordinates and the features:

$$[\hat{\epsilon}_t^{(\mathbf{x})}, \hat{\epsilon}_t^{(\mathbf{h})}] = \mathbf{EGNN}(\mathbf{z}_t^{(\mathbf{x})}, \mathbf{z}_t^{(\mathbf{h})}, t/T) - [\mathbf{z}_t^{(\mathbf{x})}, \mathbf{0}] \tag{8}$$

The subtraction $[\mathbf{z}_t^{(\mathbf{x})}, \mathbf{0}]$ ensures that the latent variable $\mathbf{z}_t$ lie at the zero center of gravity, maintaining the translation equivariance constraints.

Gradient Guidance Implementation

For the gradient guidance of the diffusion model, we employ the GeoGuide[3] model for the forward guidance, which incorporates the gradient into the latent variable $\mathbf{z}_t$ at step t:

$$\mathbf{z}_t^{(i)} = \mathbf{z}_t^{(i)} + \lambda \cdot A(\nabla_{\mathbf{z}_t} f(\mathbf{z}_t)) \tag{9}$$

where the normalized gradient term is defined as:

$$A(\nabla_{\mathbf{z}_t} f(\mathbf{z}_t)) = \frac{\sqrt{D}}{T} \cdot \frac{\nabla_{\mathbf{z}_t} f(\mathbf{z}_t)}{||\nabla_{\mathbf{z}_t} f(\mathbf{z}_t)||} \tag{10}$$

with D representing the dimensionality of the latent space and T the total number of diffusion steps. This normalization ensures a consistent and bounded influence of the guidance term throughout the reverse denoising process.

Following the forward pass, we also employ backward guidance,[4] which performs m steps of gradient descent on the latent variable at each denoising step to reinforce the influence of the guidance term. Each gradient descent update is given by:

$$A_{s,i} = \frac{\sqrt{D}}{T} \cdot \frac{\nabla_{\mathbf{z}_s} f(\hat{\mathbf{z}}s)}{|\nabla \mathbf{z}_s f(\hat{\mathbf{z}}_s)|}, \ \mathbf{z}_s \quad = \mathbf{z}s - s(t) \cdot As, i, \tag{11}$$

where $s(t)$ is a step-size function controlling the magnitude of each update.

To estimate properties of the generating molecule during the denoising process, we train the guidance model, which shares the same EGNN as the diffusion model, to minimize the mean squared error between the predicted and true property values.

$$\mathcal{L}_{\text{guidance}} = \mathbb{E}_{t\sim\mathcal{U}(0,1)} \left[\mathbb{E}_{\mathbf{z}_t\sim q(\mathbf{z}_t|\mathbf{x}_0)} \left\| f_\theta(\mathbf{z}_t, t) - y \right\|_2^2 \right] \tag{12}$$

This training setup ensures that the guidance model is capable of estimating target properties across different noise levels, enabling the computation of meaningful gradients $\nabla_{\mathbf{z}_t} f_\theta(\mathbf{z}_t, t)$ during the generative denoising process in directing the generation toward 3D molecules with target properties.

S2: 3D Chemical Structure Database

To support the development of reliable and broadly applicable pre-trained diffusion models, we compile a large and diverse chemical database of 3D molecular structures from multiple publicly available and in-house sources. A summary of these databases is provided in Table S1. For drug-like and biology-related molecules, we include the QMug[5] and GEOM[6] databases. Additionally, we incorporate our in-house FORMED[7] database, which contains synthesizable molecules extracted from the Cambridge Structural Database (CSD), as well as the OSCAR[8] database, specifically tailored for organocatalysts. Lastly, we integrate COMPAS1[9] for polycyclic aromatic systems and COMPAS2[10] for hetero-polycyclic aromatic systems.

In total, this unified database of 3D molecules comprises approximately 1.7 million unique molecules across 17 element types, with an average molecular size of 53 atoms. These molecules span a broad region of chemical space, as visualized in the dimensionality reduction plot (Figure S2). The complete set of 3D molecules is stored in NumPy format and is publicly available at `https://huggingface.co/pregH/MolecularDiffusion`.

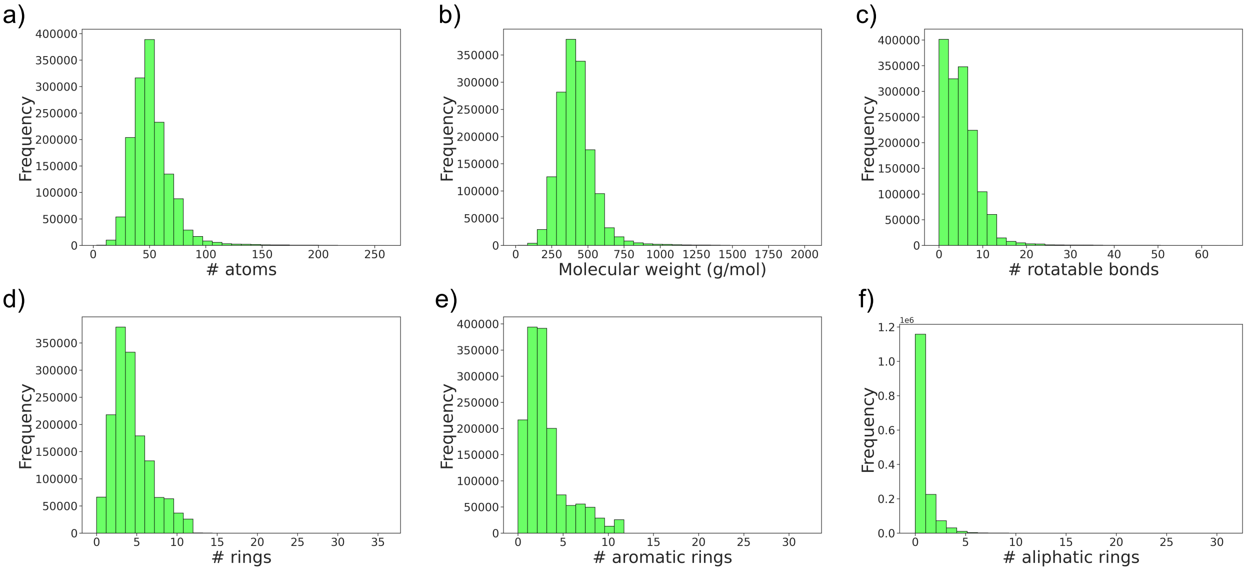

Figure S1: Distribution of properties for the 3D molecules in the compiled datasets.

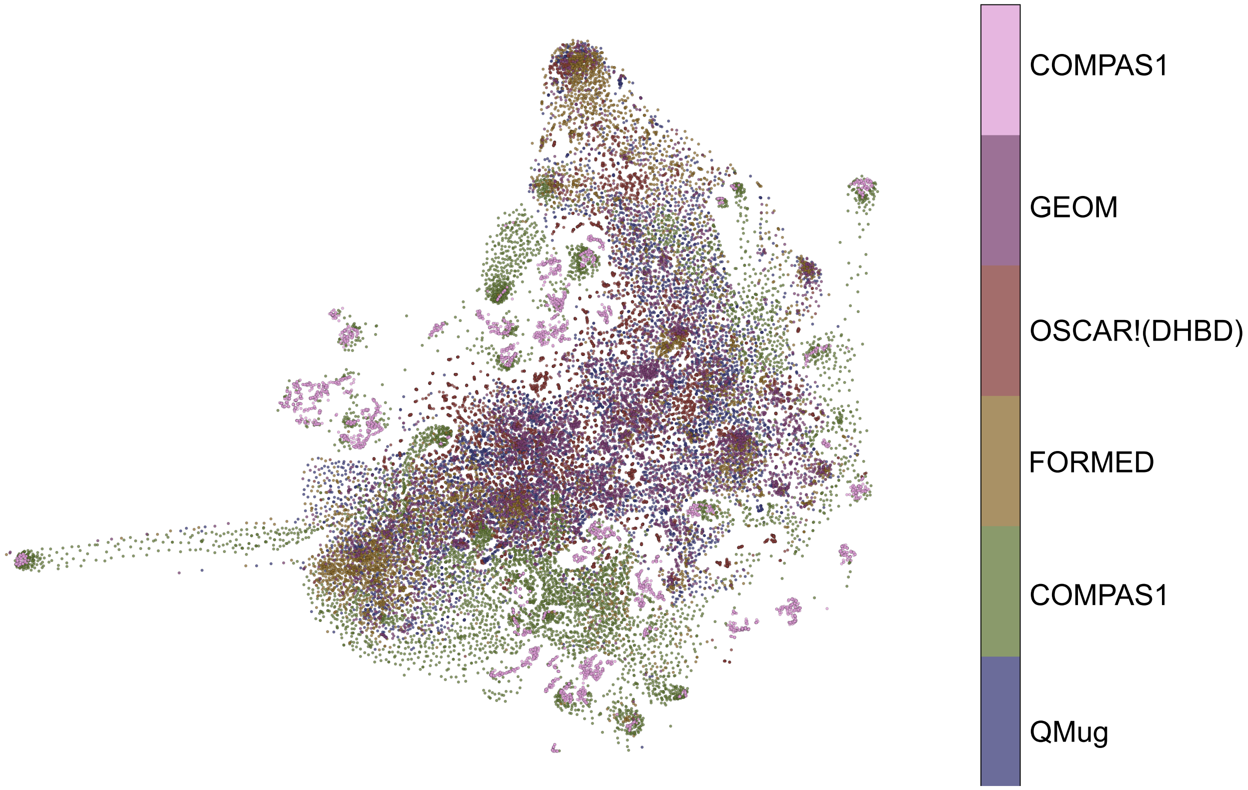

Figure S2: t-SNE plot generated from the Morgan fingerprint of the molecules in the compiled datasets.

Table S1: Summary of chemical structure databases used for pretraining the diffusion model for 3D molecule generation.

Database	Domain	Elements	Avg size	Database Size
QMug	Drug-like, biology-related	H,C,N,O,F,P,S,Cl,Br,I	~ 55 (7–228)	665k
GEOM	Drug-like, biology-related	H,B,C,N,O,F,Al,Si,P,S,Cl,As,Br,I,Hg,Bi	~ 44 (3–181)	256k
FORMED	Synthesizable molecules from CSD	H,B,C,N,O,F,Si,P,S,Cl,As,Se,Br	~ 44 (4–260)	112k
OSCAR!(DHBD)	Dual-hydrogen-bond donors organocatalysts	H, C, N, O, F, Si, P, S, Cl, Br, I	~55 (11–162)	500k
COMPAS-1	Polycyclic Aromatic Systems	H,C	~70 (12-72)	34k
COMPAS-2	Hetero-Polycyclic Aromatic Systems	H,C,B,N,O,S	~46 (11-66)	156k

S3: Structure Quality Metrics

1. **Chemical validity (%):** This metric quantifies the proportion of generated molecules in which all atoms adhere to the valency rule.

2. **Connected (%):** This metric determines the proportion of generated 3D molecules that are a single, connected entity rather than disjointed fragments.

3. **Chemically valid and connected (%):** This metric considers both chemical validity and connectivity of the molecule.

   In addition to these basic metrics, we evaluate the quality of 3D molecules using the PoseBusters sanity checks[11] as follows:

4. **Bond lengths/angles**: Fraction of molecules in which all bond lengths and angles fall within 0.75 to 1.25 times the bounds defined by distance geometry.

5. **Ring flat**: Fraction of molecules in which all atoms in 5- or 6-membered aromatic rings lie within 0.25 Å of their best-fit plane.

6. **Double bond flatness**: Fraction of molecules in which all atoms involved in aliphatic carbon-carbon double bonds, along with their four neighboring atoms, lie within 0.25 Å of the closest shared plane.

7. **No steric clash**: Fraction of molecules with no non-bonded atom pairs closer than 0.8 times the distance geometry lower bound.

8. **Internal energy**: Fraction of molecules whose calculated energy does not exceed 100 times the mean energy of an ensemble of 50 conformations generated from the input structure. Conformations are generated with ETKDGv3, energies computed using UFF[12] in RDKit,[13] and structures are relaxed with up to 200 UFF iterations.

   We also assess deviations from optimized geometries:

9. **Average RMSD (Å):** This metric quantifies the discrepancy between generated structures and their corresponding relaxed geometries by computing the root-mean-square deviation (RMSD) between the initially generated structure and its optimized counterpart at the GFN2-xTB[14] level.

10. **Intact chemical topology (%):** This metric evaluates whether the molecular connectivity remains unchanged after geometry optimization at the GFN2-xTB level. Retaining the same connectivity is essential, as the chemical identity of a molecule must be preserved upon relaxation; otherwise, the generated structure corresponds to an unstable or chemically implausible species, limiting their relevance.

    Diversity within the generated set is crucial to avoid redundancy and to ensure that the generative model explores chemical space effectively.

11. **Uniqueness:** For valid and connected molecules, uniqueness is defined as the dissimilarity between generated molecules in the generated batch. Specifically, we compute the average Tanimoto similarity based on Morgan fingerprints between a molecule and others in the batch, and define uniqueness as:

$$\text{Uniqueness} = 1 - \frac{1}{(N_{\text{gen}} - 1)\,(N_{\text{gen}} - 1)} \sum_{i=1}^{N_{\text{gen}}-1} \sum_{k=1}^{N_{\text{gen}}-1} \text{Tanimoto}(M_i, M_k) \tag{13}$$

Beyond internal diversity, it is also important to check whether the model generates molecules distinct from its training data, reflecting the ability to generalize beyond just memorizing the training data.

12. **Novelty:** For valid and connected molecules, novelty is defined as the dissimilarity between a generated molecule and those in the training set. We compute the average Tanimoto similarity based on Morgan fingerprints between all generated molecules and all molecules in the training data, then define novelty as:

$$\text{Novelty} = 1 - \frac{1}{N_{\text{gen}}\, N_{\text{train}}} \sum_{i=1}^{N_{\text{gen}}} \sum_{k=1}^{N_{\text{train}}} \text{Tanimoto}\big(M_i, M_k^{\text{train}}\big) \tag{14}$$

S4: Implementation Details

Training Detail for the Pre-trained Diffusion Model

We employed the EDM with the following architectural and diffusion hyperparameters:

- **EGNN layers:** 9 layers with 196-dimensional hidden features.
- **Noise scheduler:** Second-order polynomial schedule with a total of 900 diffusion timesteps.
- **Noise precision:** 1×10^{-5}, controlling the terminal noise level.

The training hyperparameters were configured as follows:

- **Optimizer:** Adam with a learning rate of 1×10^{-4}, $\beta_1 = 0.9$, $\beta_2 = 0.999$.
- **Learning rate scheduler:** Cosine annealing with a minimum learning rate of 1×10^{-6}.
- **Batch size:** 64 molecules.
- **Gradient clipping:** Dynamically decrease the maximum gradient norm set, with the initial value of 3000.
- **Weight decay:** 1×10^{-12}

- **Exponential moving average (EMA):** Applied to model weights with a decay factor of 0.999.

We first trained the EDM on the concatenated QM9/VQM24 dataset, using an 80/10/10 split for training, validation, and test sets, respectively, over a total of 200 epochs. For each curriculum learning stage of PCL and HCL, we fine-tuned the model for 150 epochs, keeping all training hyperparameters the same as above except for the learning rate, which was halved at each stage.

For SPL, we fine-tuned the model for 450 epochs on the full GEOM dataset using the same training configuration.

Finally, starting from the EDM model trained with HCL on GEOM, we fine-tuned it on our compiled target database, again keeping all training hyperparameters fixed, except for the learning rate, which was set to 5×10^{-5}.

All training experiments were conducted on four NVIDIA H100 GPUs. For inference, 3D molecular structures were generated using the trained diffusion models on a single NVIDIA RTX 4090 GPU.

Training Detail for the Downstream Diffusion Models

We adapted the pre-trained diffusion model to be the IFLP diffusion model, and the conditioned model in the property-directed generation, with the following training hyperparameters:

The training hyperparameters were configured as follows:

- **Number of epochs:** 200
- **Optimizer:** Adam with a learning rate of 1×10^{-4}, $\beta_1 = 0.9$, $\beta_2 = 0.999$.
- **Learning rate scheduler:** Cosine annealing with a minimum learning rate of 1×10^{-6}.
- **Batch size:** 32 molecules.

- **Gradient clipping:** Dynamically decrease the maximum gradient norm set, with the initial value of 3000.

- **Weight decay:** 1×10^{-12}

- **Exponential moving average (EMA):** Applied to model weights with a decay factor of 0.0.

As in the earlier experiments, all training was conducted on 1 NVIDIA H100 GPU and the interference with a single NVIDIA RTX 4090 GPU.

Training Detail for the Guidance Model

Using a single NVIDIA RTX 4090 GPU, we trained a 5-layer EGNN with 512-dimensional hidden features on the S_1/T_1 energy labels in the FORMED database. The training hyperparameters were configured as follows:

- **Number of epochs:** 200

- **Optimizer:** Adam with a learning rate of 2×10^{-4}, $\beta_1 = 0.9$, $\beta_2 = 0.999$.

- **Learning rate scheduler:** Reduce-on-Plateau scheduler with a patience of 20 epochs and a minimum learning rate of 1×10^{-6} and factor value of 0.7.

- **Batch size:** 64 molecules

S5: Additional Results for 3D Molecular Generation Generation with Diffusion Models

Table S2: Evaluation of 1000 generated 3D chemical structures generated with the EDM trained with different strategies, assessed by Posebusters.[11] Values are reported as mean ± standard deviation over three trials.

**Model**	Bond angles	Bond lengths	Ring flat	Double bond flat	No steric clash	Internal energy
**GEOM database**						
EDM	10.1 ± 0.7	11.4 ± 0.6	17.9 ± 0.3	17.8 ± 0.2	6.5 ± 0.5	9.3 ± 1.3
EDM + PCL	96.2 ± 0.4	96.2 ± 0.4	97.2 ± 0.4	95.6 ± 0.6	87.5 ± 0.4	92.0 ± 0.6
EDM + SPL	97.2 ± 0.5	97.2 ± 0.5	98.2 ± 0.5	97.2 ± 0.5	90.3 ± 0.7	94.2 ± 0.5
EDM + HCL	96.8 ± 0.8	96.8 ± 0.8	97.8 ± 0.9	97.4 ± 1.2	90.6 ± 1.3	94.9 ± 1.4
**Compiled 3D database**						
EDM + HCL	88.5 ± 0.2	85.3 ± 0.4	90.8 ± 0.1	89.8 ± 0.1	83.5 ± 0.2	84.9 ± 0.5

Table S3: Molecular quality metrics across different generative models. For each model, 5,000 molecules were generated and evaluated. "Valid and Connected" denotes the fraction of molecules that are chemically valid and consist of a single connected component. "Molecular Stability" follows the definition established in previous work[22] and is based on valency considerations. Geometric fidelity is quantified by the deviations in bond lengths (in Å), bond angles (in degrees), and torsional angles between the generated structures and their MMFF94-optimized counterparts. Results for all models except EDM-HCL, ADiT, and TABASCO were taken from Nikitin *et al.*'s work.[22]

**Model**	Valid and Connected (%)	Molecular Stability (%)	Bond length ($\times 10^{-2}$)	Bond angles	Torsions
EQGAT[15]	83.4 ± 0.9	87.8 ± 0.7	1.00 ± 0.04	1.15 ± 0.03	8.58 ± 0.11
JODO[16]	87.9 ± 0.3	94.0 ± 0.3	0.77 ± 0.01	0.83 ± 0.00	6.01 ± 0.07
Megalodon-quick[17]	90.0 ± 0.7	95.7 ± 0.6	0.66 ± 0.02	0.71 ± 0.01	5.58 ± 0.11
SemlaFlow[18]	92.0 ± 1.6	97.4 ± 1.2	3.10 ± 0.23	2.06 ± 0.17	6.05 ± 0.56
FlowMol2[19]	74.6 ± 1.0	93.8 ± 0.5	1.30 ± 0.04	1.62 ± 0.02	15.0 ± 0.30
Megalodon-flow[17]	94.8 ± 0.3	95.8 ± 0.4	2.30 ± 0.02	1.62 ± 0.02	5.58 ± 0.19
TABASCO (implemented)[20]	81.4 ± 1.1	99.2 ± 0.1	2.47 ± 0.02	2.08 ± 0.06	7.23 ± 0.31
ADiT (implemented)[21]	74.6 ± 1.7	95.5 ± 2.1	2.29 ± 0.15	2.26 ± 0.10	7.92 ± 0.41
EDM-HCL (*ours*)	75.0 ± 2.0	97.0 ± 0.3	1.74 ± 0.19	1.95 ± 0.88	6.45 ± 0.47

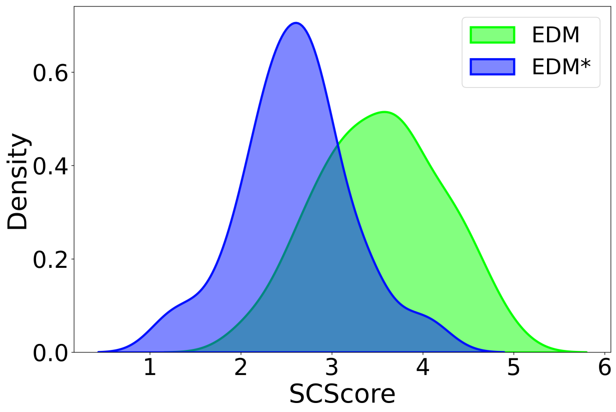

Figure S3: Kernel density estimates of the SCScore distributions for 1,000 molecules generated by models trained on the GEOM dataset: an unconditional model (EDM) and a model conditioned on SCScore (EDM*). For the EDM*, the inference was carried out using CFG with a CFG scale of 2.

S6: Additional Results for Virtual Library Design

Table S4: Summary of structural guidance diffusion performance for generating asymmetric cyclopentadienyl (Cp) ligands. For each mode, 1,000 molecules were generated. The key metrics across these trials are reported, including (1) the percentage of successful generation of chemically valid Cp ligand with the overall charge of –1, (2) the percentage of new molecules compared to BINOL-Cp(HHH), and (3) the percentage of unique molecules generated.

**Method**	**% Success**	**% Novelty**	**% Uniqueness**
Outpaint	29.88	100.00	100.00
Inpaint/d=0.3	87.00	26.00	15.25
Inpaint/d=0.4	42.70	86.83	84.80
Inpaint/d=0.5	38.50	97.53	93.64
Inpaint/d=0.6	25.50	100.00	100.00
Inpaint/d=0.7	13.30	100.00	100.00

S7: Additional Information for Designing Molecules with Target Geometries via Structural Guidance Diffusion

Table S5: Effect of different parameters on the generation performance of the outpainting methods for **INT2** structures in the catalytic CO_2 hydrogenation to formate with intramolecular frustrated Lewis pairs. The performance is assessed with 1) validity of the generated structure as **INT2** (pre-optimization validity), 2) validity of the generated structure as **INT2** following geometry optimization (post-optimization validity), and 3) the percentage of the optimized structures that are valid **INT2** and possess desirable geometrical attributes (Post-optimization hit).

Method	d (Å)	Φ	Pre-optimization Validity	Post-optimization Validity	Post-optimization hit
Direct	2.82	94.8	5.2	13.0	3.7
Direct with the extend core	2.82	94.8	9.8	36.4	10.9
Direct	2.67	33.4	5.0	15.2	3.6
Adapted	2.82	94.8	38.1	64.1	25.7
Adapted	2.67	33.4	46.7	83.8	18.6

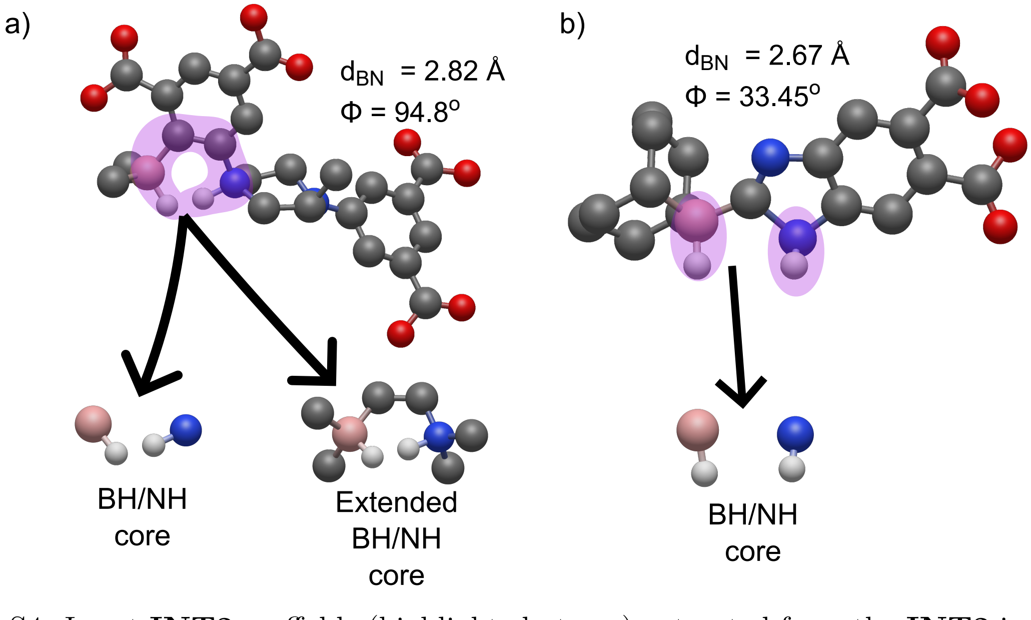

Figure S4: Input **INT2** scaffolds (highlighted atoms) extracted from the **INT2** in the IFLP dataset, employed as starting structure for the outpainting experiments. a) the scaffold with target d_{BN} and Φ, and b) the scaffold with the mean values of d_{BN} and Φ in the dataset.

Distorted Structures from the Structure-guided Generations

Structures generated with the direct outpainting methods are frequently distorted. Common issues include atoms placed unrealistically close to hydrides or protons (parts of the core scaffold), which should not form additional bonds beyond their Lewis centers (Figure S5a), as well as cases where the Lewis center itself is improperly formed due to insufficient bonding (Figure S5a-b). In addition, many generated structures appear fragmented (Figure S5c). Incorporating an extended scaffold partially mitigates these issues by improving the completeness of the Lewis center; however, the resulting Lewis substituents are often distorted or chemically invalid (Figure S5d). In contrast, the adapted approach addresses all of these problems, with the exception of ensuring full molecular connectivity (Figure S5e).

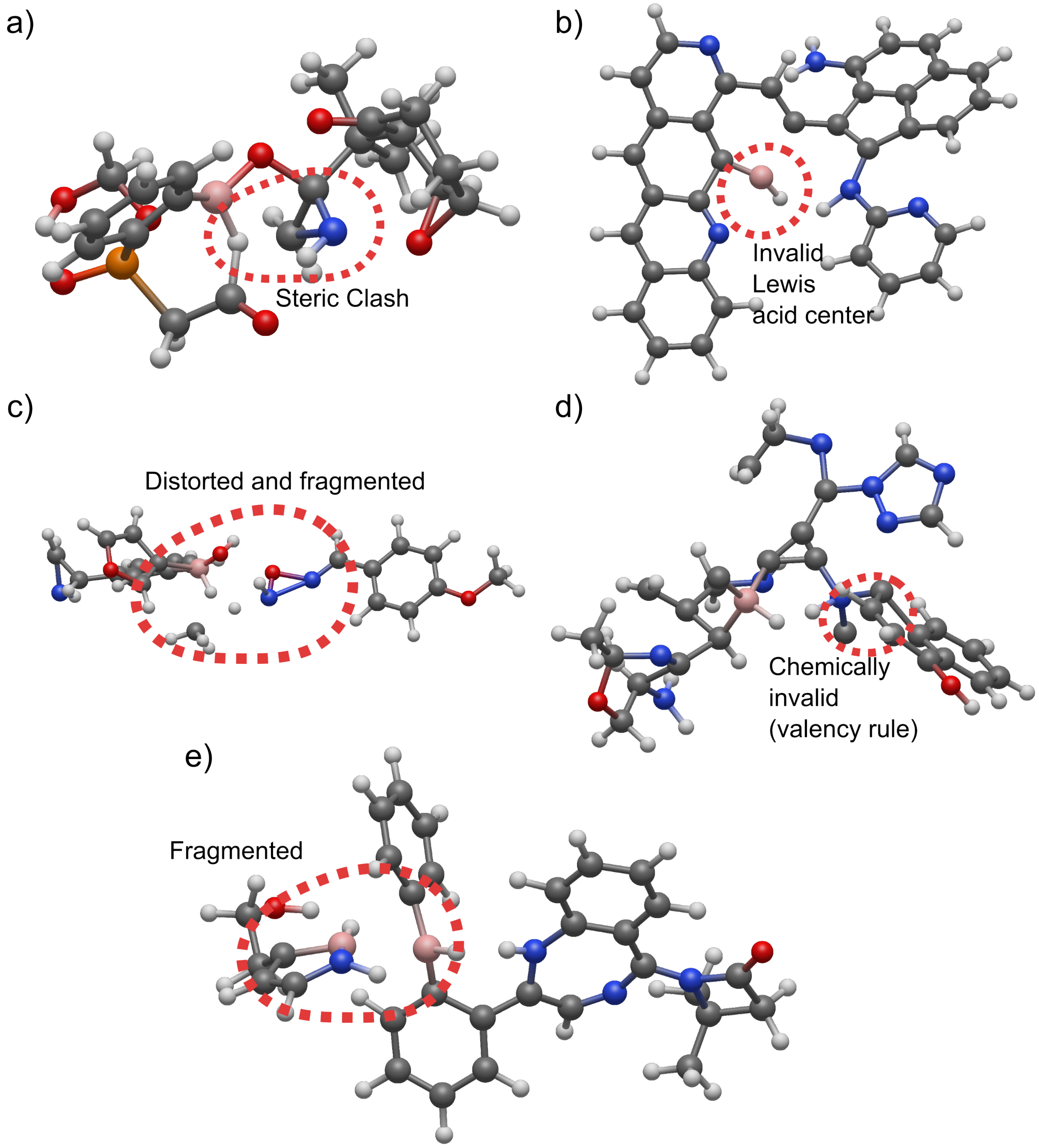

Figure S5: Examples of distorted or fragmented **INT2** structures generated using (a–c) direct outpainting with the BH/NH core, (d) direct outpainting with the extended BH/NH core, and (e) adaptive outpainting with the BH/NH core.

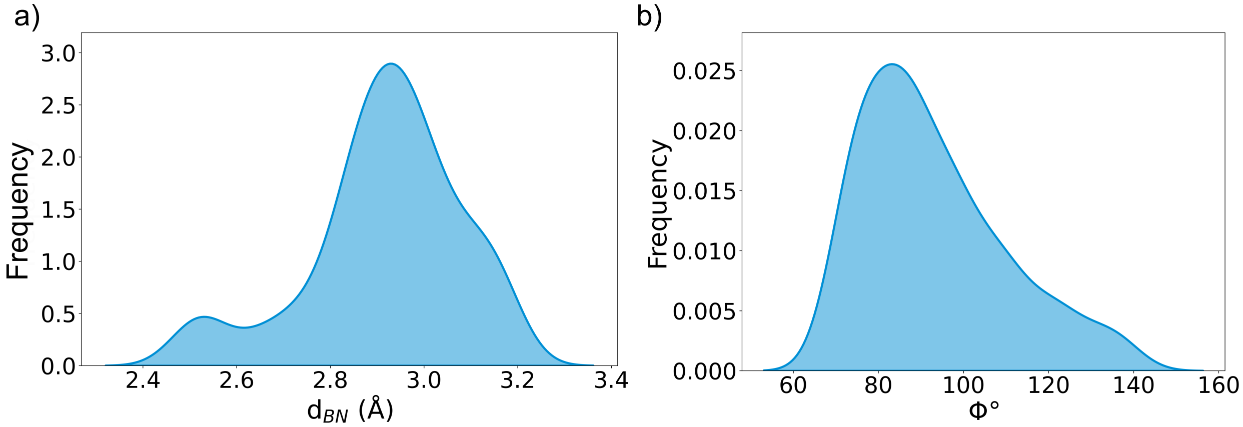

Figure S6: Kernel density estimation (KDE) plots of a) d_{BN} (Å) and $\Phi°$ for the optimized IFLP candidates generated the outpainting method.

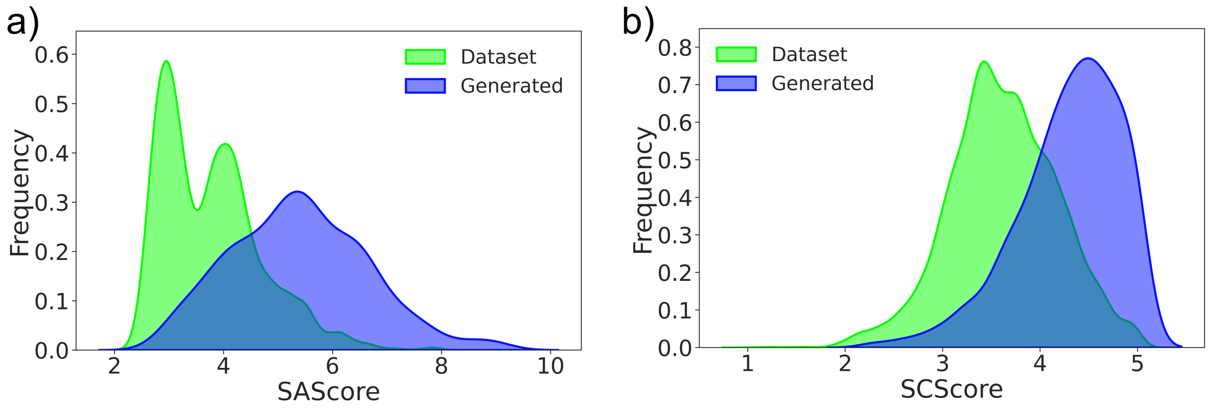

Figure S7: Kernel density histogram plots of a) SAScore and b) SCScore for molecules in **INT2** datasets and generated valid **INT2** structures.

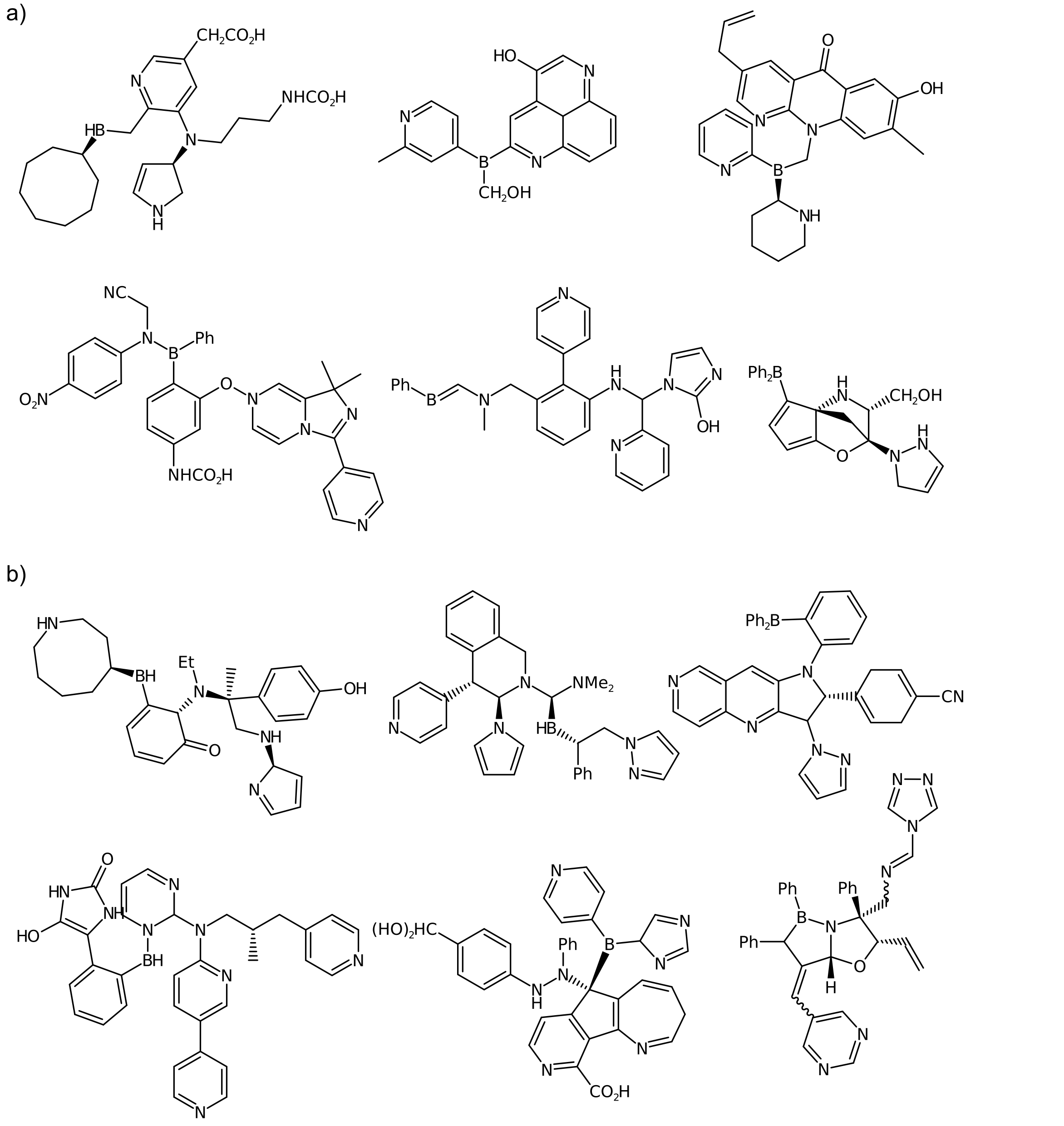

Figure S8: Representative IFLP molecules generated using molecular outpainting (a) the original EDM and (b) the EDM incorporating additional atomistic features and SCScore as the conditioning. In the latter case a CFG scale of 1.5 and an SCScore target value of 1 were used during generation.

S8: Additional Information for Designing Molecules with Target Molecular Properties via Guidance Diffusion

Singlet Fission Energy Score Function

The energy score function, following the formulation in prior work,[23] is employed to quantify how well a molecule satisfies the singlet fission (SF) energetic criteria. It is defined as the minimum signed distance to the boundary of the blue triangular region specified by the coordinates (1.5 eV, 3.8 eV), (1.9 eV, 3.8 eV), and (1.5 eV, 3.0 eV), within which the values of $E_{T_1,ve}$ and $E_{S_1,ve}$ fulfill the vertical SF energy requirements (Figure S9).

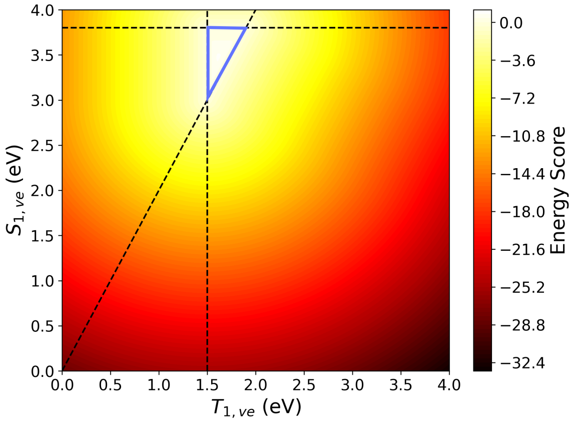

Figure S9: Energy score as a function of S_1 and T_1 for evaluating vertical (Franck-Condon) singlet fission (SF) properties. The target region, where molecules satisfy SF energetic requirements, is enclosed within the blue triangular area, corresponding to where the energy score value is positive. The dashed lines indicate the boundaries defined by the conditions: $S_{1,ve}$ - $2T_{1,ve} > 0$ eV, $S_{1,ve} = 3.8$ eV, and $T_{1,ve} = 1.5$ eV. The vertical dotted line represents the minimum $T_{1,ve}$ of 1.5 eV, a threshold for utilizing SF materials as multiexciton-generating components in Si solar cells, extrapolated from an adiabatic-vertical energy scaling relationship.[24,25] The horizontal dashed line at $S_{1,ve} = 3.8$ eV accounts for the solar energy spectrum distribution.

In an event that (S_1,T_1) lies inside the triangle, the energy score is

$$E(T_1, S_1) = \min \begin{Bmatrix} \alpha \cdot d_1(T_1, S_1), \\ \alpha \cdot d_2(T_1, S_1), \\ \alpha \cdot \beta \cdot d_3(T_1, S_1) \end{Bmatrix}$$

with $\alpha = \frac{1}{0.11871871871871865} \approx 8.42$, $\beta = \frac{1}{3}$ and distances defined as:

$$d_1 = \text{distance to vertical line } T_1 = T_{\text{cut}} = |T_1 - T_{\text{cut}}|$$
$$d_2 = \text{distance to line } S_1 = 2T_1 = \frac{|2T_1 - S_1|}{\sqrt{5}}$$
$$d_3 = \text{distance to horizontal line } S_1 = S_{\text{cut}} = |S_1 - S_{\text{cut}}|$$

If the energy pair lies outside the triangle, the score becomes a negative value reflecting the Euclidean distance to the nearest point on the triangle defined by the vertices:

$$A = (T_{\text{cut}}, 2T_{\text{cut}}, 0) = (1.5, 3.0, 0)$$
$$B = (T_{\text{cut}}, S_{\text{cut}}, 0) = (1.5, 3.8, 0)$$
$$C = \left(\frac{S_{\text{cut}}}{2}, S_{\text{cut}}, 0\right) = (1.9, 3.8, 0)$$

Let $P = (T_1, S_1, 0)$, the energy score is defined as:

$$E(S_1, T_1) = -\alpha \cdot \text{distance from } P \text{ to } \triangle ABC$$

Additional Results

Table S6: Effect of CFG scale and target value on the performance of classifier-free guidance across different evaluation metrics: percentage of molecules satisfying SF criteria, percentage of valid and connected molecules, average RMSD between generated and GFN2-xTB–optimized structures, percentage of generated molecules retaining the chemical topology of their GFN2-xTB–optimized counterparts, and the average SCScore of generated 3D molecules that can be converted to RDKit molecular objects. The row marked with an asterisk (*) corresponds to a model trained with extended atomic features (number of bonded neighbors, number of valence electrons, hybridization state, and formal charge) and SCScore conditioning.

CFG scale	Target value	%Hit	Valid and connected (%)	RMSD (Å)	Intact topology (%)	SCScore
0.0	0.0	13.9	86.4	0.75	84.2	3.6
0.5	0.0	21.1	87.6	0.66	83.7	3.8
1.0	0.0	24.1	89.9	0.65	82.5	3.8
1.5	0.0	29.1	85.9	0.66	80.2	3.9
2.0	0.0	25.7	64.5	0.71	70.6	3.8
2.5	0.0	14.8	13.9	0.54	12.4	2.9
1.5	-1.0	25.3	87.3	0.63	80.7	3.9
1.5	1.0	26.9	80.2	0.69	77.7	3.9
2.5	-10.0	10.6	58.7	0.65	23.1	3.3
1.0*	0.0*	22.1*	91.3*	0.23*	89.7*	3.4*

Table S7: Effect of guidance strength and maximum gradient norm on the performance of the gradient guidance across different evaluation metrics: percentage of molecules satisfying SF criteria, percentage of valid and connected molecules, average RMSD between generated and GFN2-xTB–optimized structures, and percentage of generated molecules retaining the chemical topology of their GFN2-xTB–optimized counterparts and the average SCScore of generated 3D molecules that can be converted to RDKit molecular objects.

Guidance strength	Maximum gradient norm	%Hit	Valid and connected (%)	RMSD Å	Intact topology (%)	SCScore
0.001	100.0	41.3	78.9	1.05	83.9	3.9
0.002	100.0	62.9	65.6	1.05	73.5	3.8
0.004	100.0	61.7	53.8	0.90	35.1	3.8
0.008	100.0	30.9	18.9	0.97	37.5	3.3
0.002	10.0	70.2	71.2	0.92	74.8	3.9
0.002	1.0	38.6	79.6	1.03	82.1	3.9
0.002	0.1	36.3	75.0	1.02	84.2	3.9

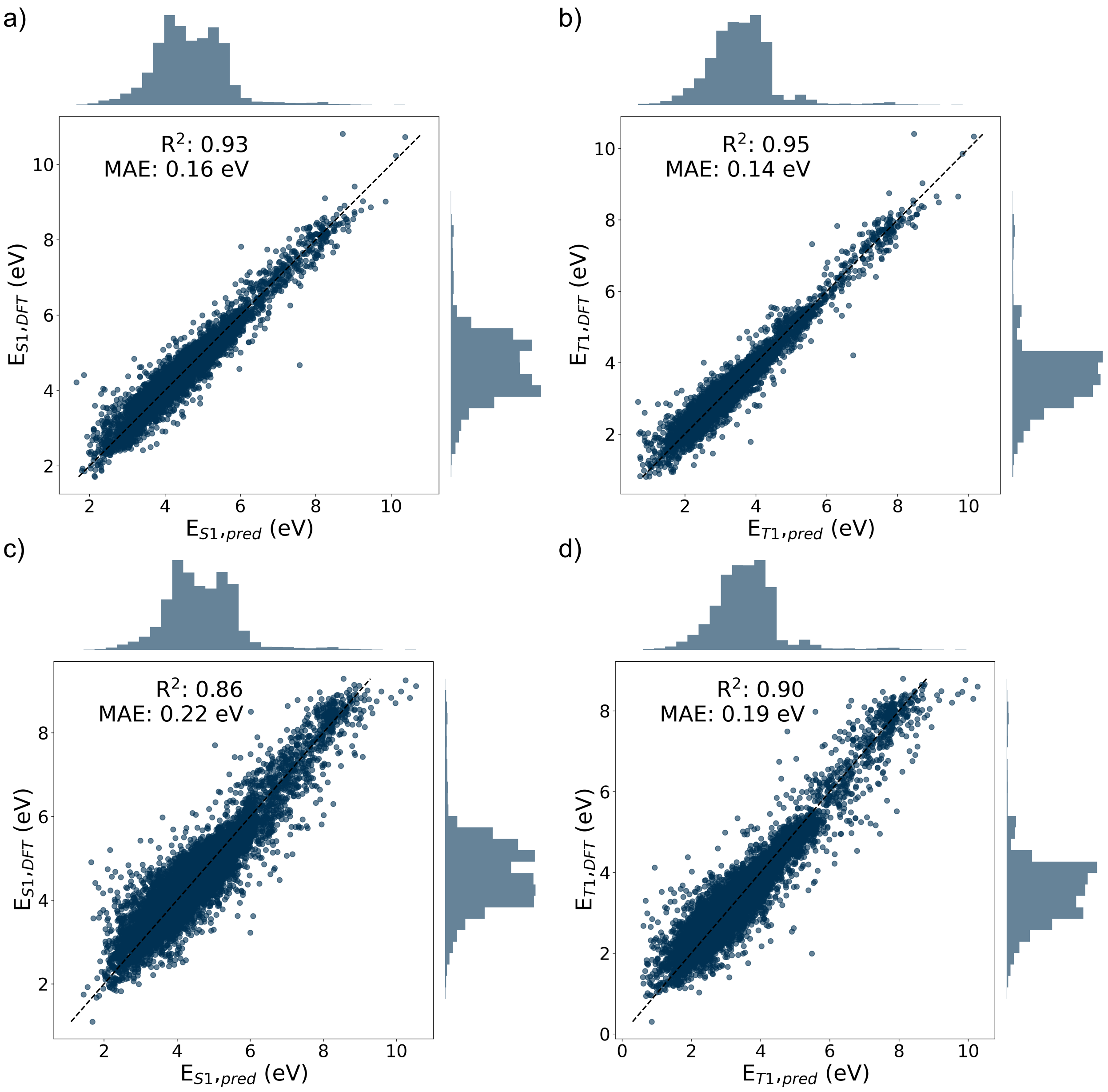

Figure S10: Correlation plots of a) $E_{S_1,\mathrm{ve}}$, b) $E_{T_1,\mathrm{ve}}$ comparing the predicted values from the regressor model with the true values for molecules in the test set. Correlation plots of c) $E_{S_1,\mathrm{ve}}$, d) $E_{T_1,\mathrm{ve}}$ comparing the predicted values from the guidance model with the true values for molecules in the test set.

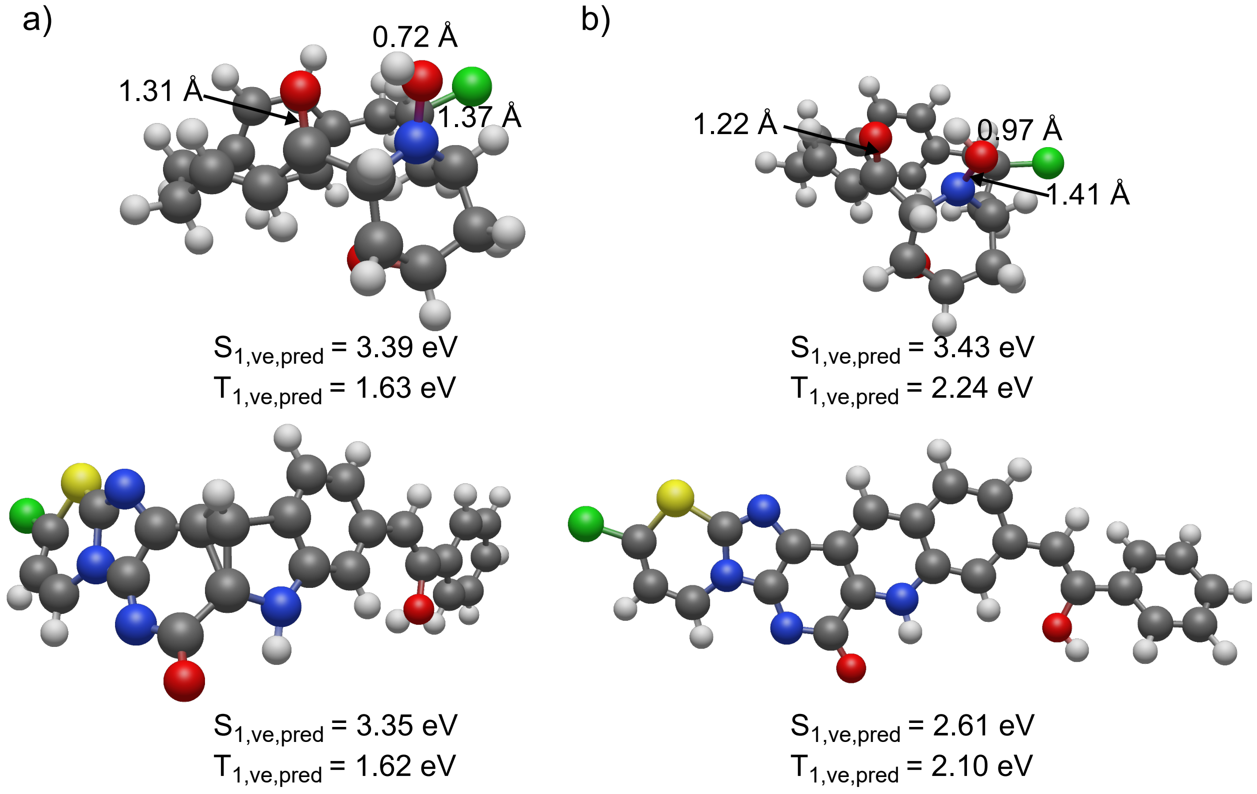

Figure S11: Examples of pre-optimization hit molecules generated with gradient guidance mode that fail to retain SF properties following geometry optimization at GFN2-xTB.

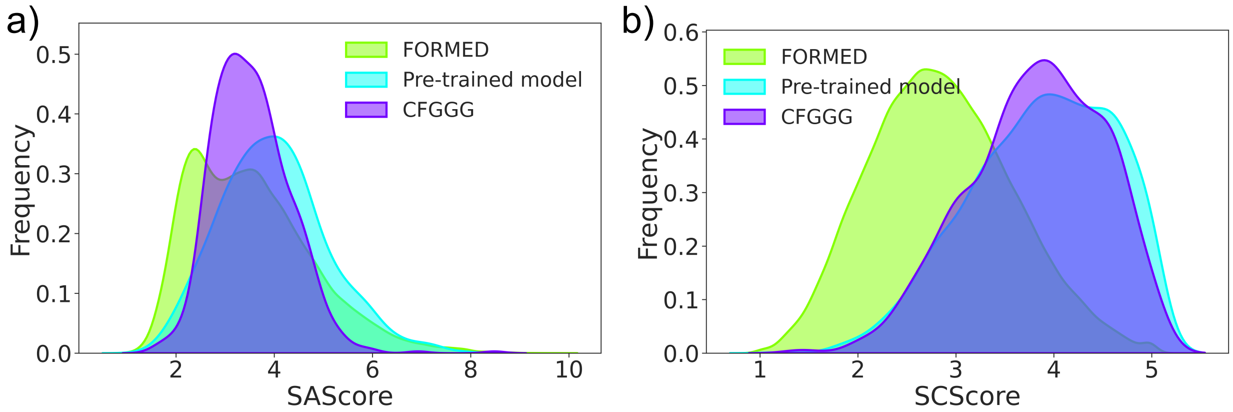

Figure S12: Kernel density histogram plots of a) SAScore and b) SCScore for molecules in the FORMED dataset and generated molecules from the pre-trained diffusion model and the hybrid guidance method, targeting SF properties.

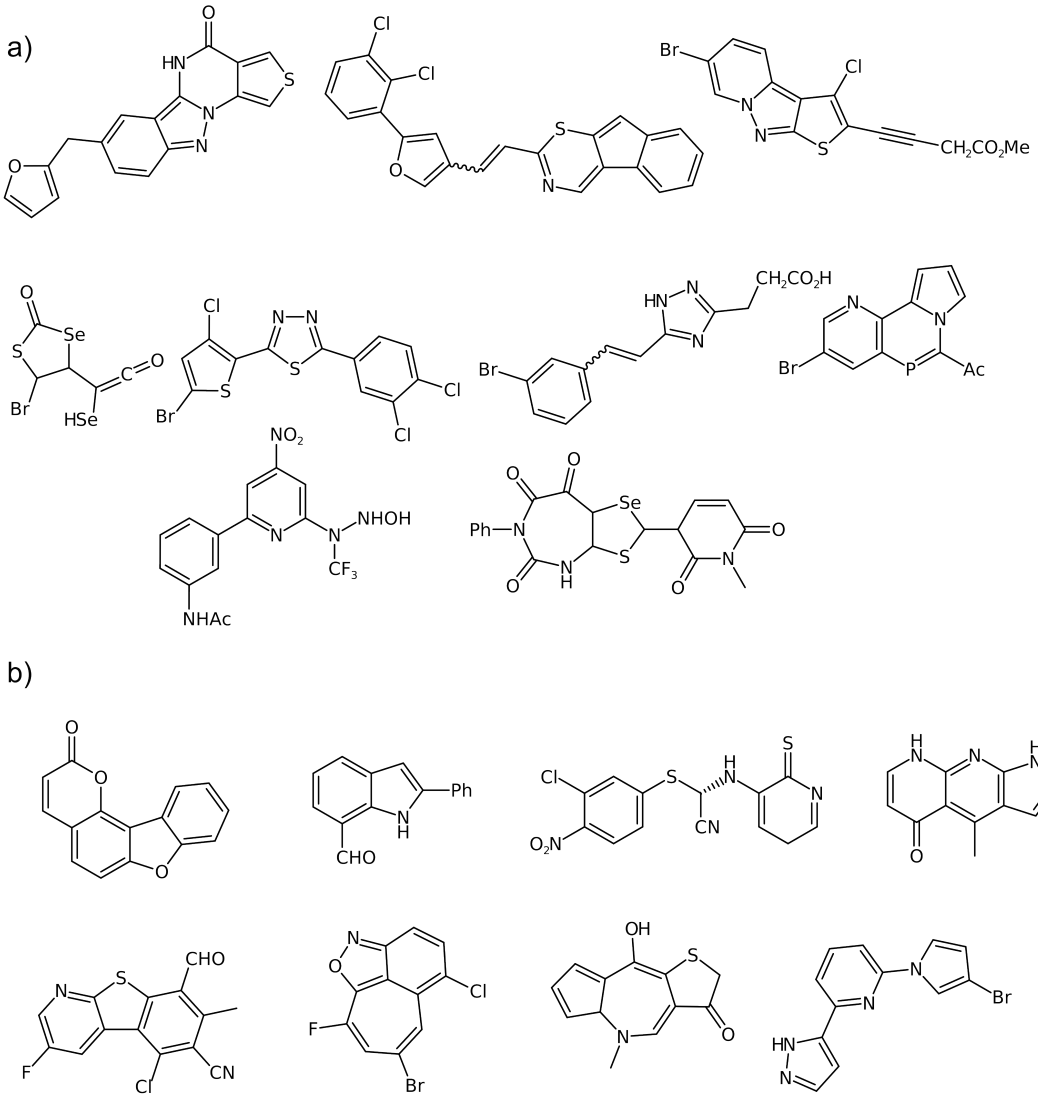

Figure S13: Representative molecules generated using CFG–based property-guided sampling toward singlet fission properties with (a) the original EDM and (b) the EDM incorporating additional atomistic features. In both cases, a CFG scale of 1.5 and an energy target value of 0 were used during generation; in (b), SCScore conditioning with a target value of 1 was additionally applied.

Table S8: Summary of diffusion guidance modes for goal-directed generations. For each mode, 1,000 molecules were generated in three independent trials. The mean and standard deviation of key metrics across these trials are reported. These include the (1) percentage of valid and fully connected molecules, (2) RMSD (Å) measured between the generated geometries and the optimized counterparts, (3) percentage of molecules with intact topology (i.e., unchanged connectivity matrices before and after optimization), (4) percentage of pre-optimization hits that become post-optimization misses, (5) percentage of pre-optimization misses that become post-optimization hits, (6) percentage of molecules that are hits both before and after optimization, (7) uniqueness score, and (8) novelty score

Guidance mode	%Valid and connected	RMSD (Å)	%Intact topology	%pre-opt hit → post-opt miss	%pre-opt miss → post-opt hit	%both hit	Uniquenes	Novelty
GG	65.6±2.1	1.02±0.02	71.5 ±5.7	51.9±3.0	2.0±0.1	11.6±2.4	0.78±0.02	0.68±0.01
CFG	89.1±3.0	0.53±0.02	85.6±0.8	4.5±0.6	5.8±0.4	23.2±1.1	0.74±0.01	0.69±0.01
CFG/GG	84.7±1.3	0.53±0.02	84.1±1.4	39.8±3.5	2.9±0.1	41.0±3.2	0.74±0.00	0.68,0.00

S9: Ablation Study of Diffusion Models.

Table S9: Hyperparameters for the equivariant diffusion models. Base node features consist of one-hot encoding of atom types and atomic numbers. Extra node features are the number of bonded neighbors, the number of valence electrons, hybridization, and formal charge.

Hyperparameters	EDM-0	EDM-1	EDM-2	EDM-3	EDM-4	EDM-0-aug
Number of layers	9	12	6	9	9	9
Number of hidden features	256	356	192	256	256	256
Attentions	✓	✓	✓	×	✓	✓
Noise scheduler	polynomial	polynomial	polynomial	polynomial	cosine	polynomial
Extra Node Features	×	×	×	×	×	✓
Number of parameters	5.5M	9.7M	3.8M	5.5M	5.5M	5.6M

https://zenodo.org/records/19511401

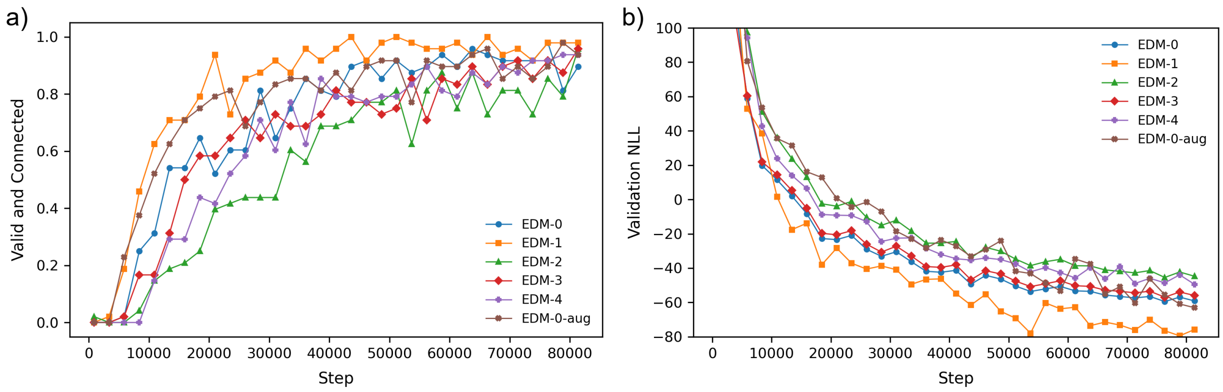

Figure S14: Learning curves of different equivariant diffusion models. (a) Fraction of generated molecules that are valid and form a single connected component, evaluated during training; (b) validation loss.

To assess the impact of architectural choices and model complexity on performance and training behavior, we conducted an ablation study of the equivariant diffusion model on the QM9 dataset. Table S9 summarizes the architectural configurations considered, including variations in model depth, hidden feature dimensionality, attention usage, noise scheduling scheme, and the inclusion of additional atomistic features.

Increasing model capacity leads to improved generative performance and faster convergence, at the cost of higher computational expense (Figure S11, Table S10). In addition, under

Table S10: Percentage of valid and connected molecules, root-mean-square deviation (RMSD) between the generated structures and their optimized counterparts, and training time for different EDM variants. All models were trained on the QM9 dataset[26] for 80,000 iterations using an NVIDIA RTX 4090 GPU on Ubuntu 22.04.

**Model**	Valid and Connected (%)	RMSD	Training time (min)
EDM-0	91.8	0.37	52
EDM-1	95.8	0.32	107
EDM-2	80.3	0.55	39
EDM-3	92.2	0.40	48
EDM-4	86.5	0.45	53
EDM-0-aug	91.4	0.43	83

comparable architectural settings, the second-order polynomial noise scheduler consistently yields better training stability and final performance than the cosine schedule (Table S10).

Furthermore, we examine the effect of incorporating extended atomistic features, including the number of bonded neighbors, the number of valence electrons, the hybridization state, and the formal charge. Models trained with these additional features exhibit faster convergence during training (Figure S12), suggesting that enriched node representations can facilitate learning. Under the configurations considered here, the final generative performance in terms of validity, connectivity, and RMSD relative to optimized geometries is comparable to that of models trained without these additional features (Table S10).

S10: Pharmocophore-conditioned Generation

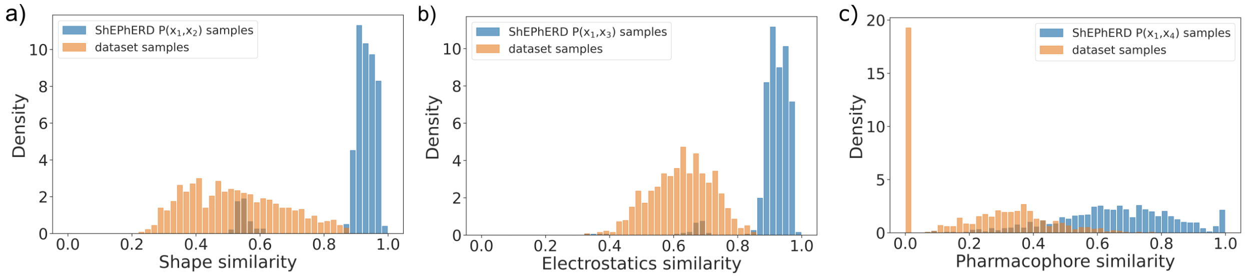

Figure S15: 3D similarity between generated and reference interaction profiles for 1,000 molecules generated unconditionally using the ShEPhERD-GDB17 pre-trained weights from Adams *et al.*:[27] (a) molecular shape, (b) electrostatic potential (ESP) surface, and (c) pharmacophore profile. Distributions show self-consistency of the generative models (generated profiles vs. computed profiles of the generated 3D molecules) overlaid with baseline similarity against randomly selected molecules (and their profiles) from ShEPhERD-GDB17.

S11: Software Implementation Details

Architecture Overview

The framework is implemented as a Python package designed for modularity, scalability, and reproducibility. It follows a layered architecture that decouples core training logic from model definitions and task-specific implementations. The system is designed to support the entire lifecycle of 3D molecular generative models, from data ingestion to training or fine-tuning, 3D molecular generation, and 3D molecule analysis.

Functional Module Organization

The application functionality is organized into distinct modules, each responsible for a specific stage of the workflow:

- **Training Module:** An engine for training 3D molecular diffusion models, as well as property regressors and guidance models.
- **Generation Module:** A generation pipeline capable of operating in multiple modes: unconditional generation, inpainting, outpainting, and conditional generation. It implements property-guided sampling algorithms, including Classifier-Free Guidance (CFG), Gradient Guidance (GG), and hybrid CFG/GG. It also supports pharmacophore-conditioned generation, as proposed in the ShEPhERD[27] model.
- **Prediction:** A module for property regression from 3D molecules as the input.
- **Analysis Module:** A comprehensive suite of evaluation tools for systematically benchmarking 3D generative models. It includes a variety of validity checks (e.g., PoseBuster, valency), geometric stability assessments against optimized conformations (energy, RMSD, geometrical differences), and chemical diversity metrics (Tanimoto-based uniqueness/novelty). MolCraftDiffusion also interfaces with

xTB, enabling geometry optimization and electronic descriptor computation (e.g., HOMO–LUMO gap, dipole moment, ionization potential, and electron affinity), and implements the 3D-coordinate-to-graph method for determining SMILES and RDKit molecular objects from generated 3D geometries. Together, these tools. This standardization facilitates the rigorous comparison of different models implemented within the package.

Data Management Infrastructure

The data layer is designed to handle multi-modal molecular datasets with varying levels of structural information. It supports multiple input specifications, allowing users to train on:

- **Geometric Data (XYZ):** Direct loading of molecular geometries from directories of .xyz files combined with metadata in .csv format for property annotation if available.
- **ASE Databases:** Native support for Atomic Simulation Environment (ASE) database files (.db).
- **Pickle files:** RDKit mol blocks with conformer information, stored as `.pkl`.
- **Pre-processed Binaries:** Fast loading of pre-processed serialized datasets stored as `.pt` or `.pkl` files, including RDKit mol blocks with conformer information.

The data module automatically handles collation based on the architecture requirements, supporting both PyTorch Geometric (PyG) Graphs (sparse batching for GNNs) and Dense Point Clouds (padded tensor batches with masks). It natively handles parsing, normalization, and featurization by interfacing with external chemistry toolkits such as RDKit,[13] Morfeus,[28] and Cosymblib.[29] This design allows chemically relevant information beyond atomic identity to be incorporated into the node representations in training diffusion models or regressor models.

Command-Line Interface and Configuration

All platform functionality is accessible through a unified command-line interface (CLI), `MolCraftDiff`, which exposes six commands covering the full modeling pipeline: `train`, `generate`, `predict`, `analyze`, `data`, and `eval-predict`. Workflows are fully specified via YAML configuration files, which define model architecture, training hyperparameters, dataset paths, and generation or evaluation parameters. No modification of the underlying source code is required to run any supported workflow, as illustrated by the following usage pattern:

```
Usage: MolCraftDiff [OPTIONS] COMMAND [ARGS]...

  A unified command-line interface for training, generation, and prediction
  with molecular diffusion models.

  Examples:
      molcraft train    configs/my_train_config.YAML
      molcraft generate configs/my_gen_config.YAML
      molcraft predict  configs/my_pred_config.YAML

Commands:
  train         Train a molecular diffusion model.
  generate      Generate molecules using a trained model.
  predict       Run property prediction on molecules.
  analyze       Analyze 3D molecular structures.
  data          Data processing utilities.
  eval-predict  Evaluate model predictions on validation/test sets.
```

To support reproducibility, YAML configuration files corresponding to each experiment

described in the manuscript are provided in the GitHub repository. This design substantially lowers the barrier to adoption for domain chemists and researchers without machine learning or software engineering expertise. Tutorials covering configuration and usage for each module are publicly available at `https://preghosh.github.io/MolCraftDiffusion/`.

Example Configuration Files

Training configuration (`train.yaml`):

```
defaults:
  - data: mol_dataset
  - tasks: diffusion
  - _self_

name: "EDM_qm9"
seed: 86
contexts: []            # specify property contexts for conditional training

trainer:
  output_path: "training_outputs/${name}"
  num_steps: 300000
  lr: 0.0001

data:
  data_type: "pointcloud"
  batch_size: 64
  dataset_name: "qm9"
  ase_db_path: "data/qm9.db"   # path to ASE database
  target_fields: ${contexts}
```

```
tasks:
  hidden_size: 256
  num_layers: 12
```

Generation configuration (`gen.yaml`):

```
defaults:
  - tasks: diffusion
  - interference: gen_unconditional
  - _self_

chkpt_directory: training_outputs/EDM_geom/  # path to trained model
atom_vocab: [H,B,C,N,O,F,Al,Si,P,S,Cl,As,Se,Br,I,Hg,Bi]
diffusion_steps: 900

interference:
  task_type: unconditional
  num_generate: 100
  batch_size: 12
  max_mol_size: 100
  output_path: ${chkpt_directory}/output    # path for generated molecules
```

All configuration files used in the experiments described in this manuscript are provided in the GitHub repository at `https://zenodo.org/records/19511401`.

Extensibility and Customization

MolCraftDiffusion is designed for straightforward extension at each layer of the pipeline:

- **Featurization:** Additional atomic or molecular features can be incorporated into node representations via the data module.

- **Backbone networks:** New neural network architectures can be registered and used as drop-in replacements within the training module.

- **Diffusion frameworks and generation strategies:** New diffusion formulation or specialized conditional generation schemes can be defined within the training and/or generation modules, respectively.

- **Evaluation metrics:** New metrics for assessing the quality or specific physicochemical properties of generated 3D molecules can be added to the analysis module.

References

(1) Hoogeboom, E.; Satorras, V. G.; Vignac, C.; Welling, M. Equivariant diffusion for molecule generation in 3d. International conference on machine learning. 2022; pp 8867–8887.

(2) Song, Y.; Durkan, C.; Murray, I.; Ermon, S. Maximum likelihood training of score-based diffusion models. *Adv. Neural Inf. Process Syst.* **2021**, *34*, 1415–1428.

(3) Poleski, M.; Tabor, J.; Spurek, P.; others GeoGuide: Geometric guidance of diffusion models. *arXiv preprint arXiv:2407.12889* **2024**,

(4) Bansal, A.; Chu, H.-M.; Schwarzschild, A.; Sengupta, S.; Goldblum, M.; Geiping, J.; Goldstein, T. Universal guidance for diffusion models. Proceedings of the IEEE/CVF Conference on Computer Vision and Pattern Recognition. 2023; pp 843–852.

(5) Neeser, R. M.; Isert, C.; Stuyver, T.; Schneider, G.; Coley, C. W. QMugs 1.1: Quantum mechanical properties of organic compounds commonly encountered in reactivity datasets. *Chem. Data Collect.* **2023**, *46*, 101040.

(6) Axelrod, S.; Gómez-Bombarelli, R. GEOM, energy-annotated molecular conformations for property prediction and molecular generation. *Sci. Data.* **2022**, *9*, 185.

(7) Blaskovits, J. T.; Laplaza, R.; Vela, S.; Corminboeuf, C. Data-Driven Discovery of Organic Electronic Materials Enabled by Hybrid Top-Down/Bottom-Up Design. *Adv. Mater.* **2024**, *36*, 2305602.

(8) Gallarati, S.; Gerwen, P. v.; Laplaza, R.; Vela, S.; Fabrizio, A.; Corminboeuf, C. OSCAR: an extensive repository of chemically and functionally diverse organocatalysts. *Chem. Sci.* **2022**, *13*, 13782–13794.

(9) Wahab, A.; Pfuderer, L.; Paenurk, E.; Gershoni-Poranne, R. The COMPAS Project:

A Computational Database of Polycyclic Aromatic Systems. Phase 1: cata-Condensed Polybenzenoid Hydrocarbons. *J. Chem. Inf. Model.* **2022**, *62*, 3704–3713.

(10) Mayo Yanes, E.; Chakraborty, S.; Gershoni-Poranne, R. COMPAS-2: a dataset of cata-condensed hetero-polycyclic aromatic systems. *Sci. Data.* **2024**, *11*, 97.

(11) Buttenschoen, M.; M. Morris, G.; M. Deane, C. PoseBusters: AI-based docking methods fail to generate physically valid poses or generalise to novel sequences. *Chem. Sci.* **2024**, *15*, 3130–3139.

(12) Rappe, A. K.; Casewit, C. J.; Colwell, K. S.; Goddard, W. A.; Skiff, W. M. UFF, a full periodic table force field for molecular mechanics and molecular dynamics simulations. *J. Am. Chem. Soc.* **1992**, *114*, 10024–10035.

(13) RDKit: Open-source cheminformatics. `http://www.rdkit.org`.

(14) Bannwarth, C.; Ehlert, S.; Grimme, S. GFN2-xTB—An accurate and broadly parametrized self-consistent tight-binding quantum chemical method with multipole electrostatics and density-dependent dispersion contributions. *J. Chem. Theory Comput.* **2019**, *15*, 1652–1671.

(15) Le, T.; Cremer, J.; Noé, F.; Clevert, D.-A.; Schütt, K. Navigating the design space of equivariant diffusion-based generative models for de novo 3d molecule generation. *arXiv preprint arXiv:2309.17296* **2023**, (accessed 2026-05-15).

(16) Huang, H.; Sun, L.; Du, B.; Lv, W. Learning joint 2d & 3d diffusion models for complete molecule generation. *arXiv preprint arXiv:2305.12347* **2023**,

(17) Reidenbach, D.; Nikitin, F.; Isayev, O.; Paliwal, S. G. Applications of Modular Co-Design for De Novo 3D Molecule Generation. *Digit. Discov.* **2026**,

(18) Irwin, R.; Tibo, A.; Janet, J. P.; Olsson, S. Efficient 3d molecular generation with flow matching and scale optimal transport. ICML 2024 AI for Science Workshop. 2024.

(19) Dunn, I.; Koes, D. R. Mixed continuous and categorical flow matching for 3d de novo molecule generation. *ArXiv* **2024**, arXiv–2404.

(20) Vonessen, C.; Harris, C.; Cretu, M.; Lio, P. TABASCO: A Fast, Simplified Model for Molecular Generation with Improved Physical Quality. *arXiv preprint arXiv:2507.00899* **2025**,

(21) Joshi, C. K.; Fu, X.; Liao, Y.-L.; Gharakhanyan, V.; Miller, B. K.; Sriram, A.; Ulissi, Z. W. All-atom diffusion transformers: Unified generative modelling of molecules and materials. *arXiv preprint arXiv:2503.03965* **2025**, (accessed 2026-05-15).

(22) Nikitin, F.; Dunn, I.; Ryan Koes, D.; Isayev, O. GEOM-drugs revisited: toward more chemically accurate benchmarks for 3D molecule generation. *Digit. Discov.* **2025**, *4*, 3282–3291.

(23) Schaufelberger, L.; Blaskovits, J. T.; Laplaza, R.; Jorner, K.; Corminboeuf, C. Inverse Design of Singlet-Fission Materials with Uncertainty-Controlled Genetic Optimization. *Angew. Chem. Int. Ed.* **2025**, *64*, e202415056.

(24) Blaskovits, J. T.; Fumanal, M.; Vela, S.; Cho, Y.; Corminboeuf, C. Heteroatom oxidation controls singlet–triplet energy splitting in singlet fission building blocks. *Chem. Commun.* **2022**, *58*, 1338–1341.

(25) Blaskovits, J. T.; Fumanal, M.; Vela, S.; Corminboeuf, C. Designing Singlet Fission Candidates from Donor–Acceptor Copolymers. *Chem. Mater.* **2020**, *32*, 6515–6524.

(26) Ramakrishnan, R.; Dral, P. O.; Rupp, M.; Von Lilienfeld, O. A. Quantum chemistry structures and properties of 134 kilo molecules. *Sci. Data* **2014**, *1*, 1–7.

(27) Adams, K.; Abeywardane, K.; Fromer, J. C.; Coley, C. W. ShEPhERD: Diffusing Shape, Electrostatics, and Pharmacophores for Bioisosteric Drug Design. International Conference on Learning Representations (ICLR). 2025; (accessed 2026-05-15).

(28) Jorner, K.; Turcani, L. kjelljorner/morfeus: v0.7.2. 2022; https://doi.org/10.5281/zenodo.7017599.

(29) Alemany, P.; Bernuz, E.; Carreras, A.; Llunell, M. Cosymlib: a Python library for continuous symmetry measures. 2021; https://doi.org/10.5281/zenodo.4925767.
